# The dual face of microglia (M1/M2) as a potential target in the protective effect of nutraceuticals against neurodegenerative diseases

**DOI:** 10.3389/fragi.2023.1231706

**Published:** 2023-09-06

**Authors:** Samar F. Darwish, Abdullah M. M. Elbadry, Amir S. Elbokhomy, Ghidaa A. Salama, Rania M. Salama

**Affiliations:** ^1^ Pharmacology and Toxicology Department, Faculty of Pharmacy, Badr University in Cairo (BUC), Cairo, Egypt; ^2^ Faculty of Pharmacy, Badr University in Cairo (BUC), Cairo, Egypt; ^3^ Nanotechnology Research Center (NTRC), The British University in Egypt (BUE), El-Sherouk City, Egypt; ^4^ Pharmacology and Toxicology Department, Faculty of Pharmacy, Misr International University, Cairo, Egypt

**Keywords:** microglia, M1/M2 pathway, neurodegeneration, nutraceuticals, aging diseases

## Abstract

The pathophysiology of different neurodegenerative illnesses is significantly influenced by the polarization regulation of microglia and macrophages. Traditional classifications of macrophage phenotypes include the pro-inflammatory M1 and the anti-inflammatory M2 phenotypes. Numerous studies demonstrated dynamic non-coding RNA modifications, which are catalyzed by microglia-induced neuroinflammation. Different nutraceuticals focus on the polarization of M1/M2 phenotypes of microglia and macrophages, offering a potent defense against neurodegeneration. Caeminaxin A, curcumin, aromatic-turmerone, myricetin, aurantiamide, 3,6′-disinapoylsucrose, and resveratrol reduced M1 microglial inflammatory markers while increased M2 indicators in Alzheimer’s disease. Amyloid beta-induced microglial M1 activation was suppressed by andrographolide, sulforaphane, triptolide, xanthoceraside, piperlongumine, and novel plant extracts which also prevented microglia-mediated necroptosis and apoptosis. Asarone, galangin, baicalein, and *a*-mangostin reduced oxidative stress and pro-inflammatory cytokines, such as interleukin (IL)-1, IL-6, and tumor necrosis factor-alpha in M1-activated microglia in Parkinson’s disease. Additionally, myrcene, icariin, and tenuigenin prevented the nod-like receptor family pyrin domain-containing 3 inflammasome and microglial neurotoxicity, while *a*-cyperone, citronellol, nobiletin, and taurine prevented NADPH oxidase 2 and nuclear factor kappa B activation. Furthermore, other nutraceuticals like plantamajoside, swertiamarin, urolithin A, kurarinone, Daphne genkwa flower, and *Boswellia serrata* extracts showed promising neuroprotection in treating Parkinson’s disease. In Huntington’s disease, elderberry, curcumin, iresine celosia, *Schisandra chinensis*, gintonin, and pomiferin showed promising results against microglial activation and improved patient symptoms. Meanwhile, linolenic acid, resveratrol, *Huperzia serrata*, icariin, and baicalein protected against activated macrophages and microglia in experimental autoimmune encephalomyelitis and multiple sclerosis. Additionally, emodin, esters of gallic and rosmarinic acids, Agathisflavone, and sinomenine offered promising multiple sclerosis treatments. This review highlights the therapeutic potential of using nutraceuticals to treat neurodegenerative diseases involving microglial-related pathways.

## 1 Introduction

### 1.1 Microglia pathway

Microglia are specialized macrophages, that constitute the primary central nervous system (CNS) innate immune cells. They are the first glial cells that enter the CNS during prenatal development. They represent approximately 10%–15% of all CNS cells. Microglia control CNS homeostasis at rest by eliminating pathogens and cell residue through phagocytic activity. Resting microglia become activated and generate inflammatory mediators, thus providing neurons protection and defense against infections. In addition to supporting the CNS, they are linked to the development of many inflammatory and neurodegenerative disorders (Colonna and Butovsky, 2017; Saitgareeva et al., 2020). Depending on the activation, microglia are separated into two categories: M1 microglia, which stimulates inflammation and neurotoxicity, and M2 microglia, which stimulates anti-inflammatory and neuroprotective effects (Qin et al., 2023).

**FIGURE 1:**
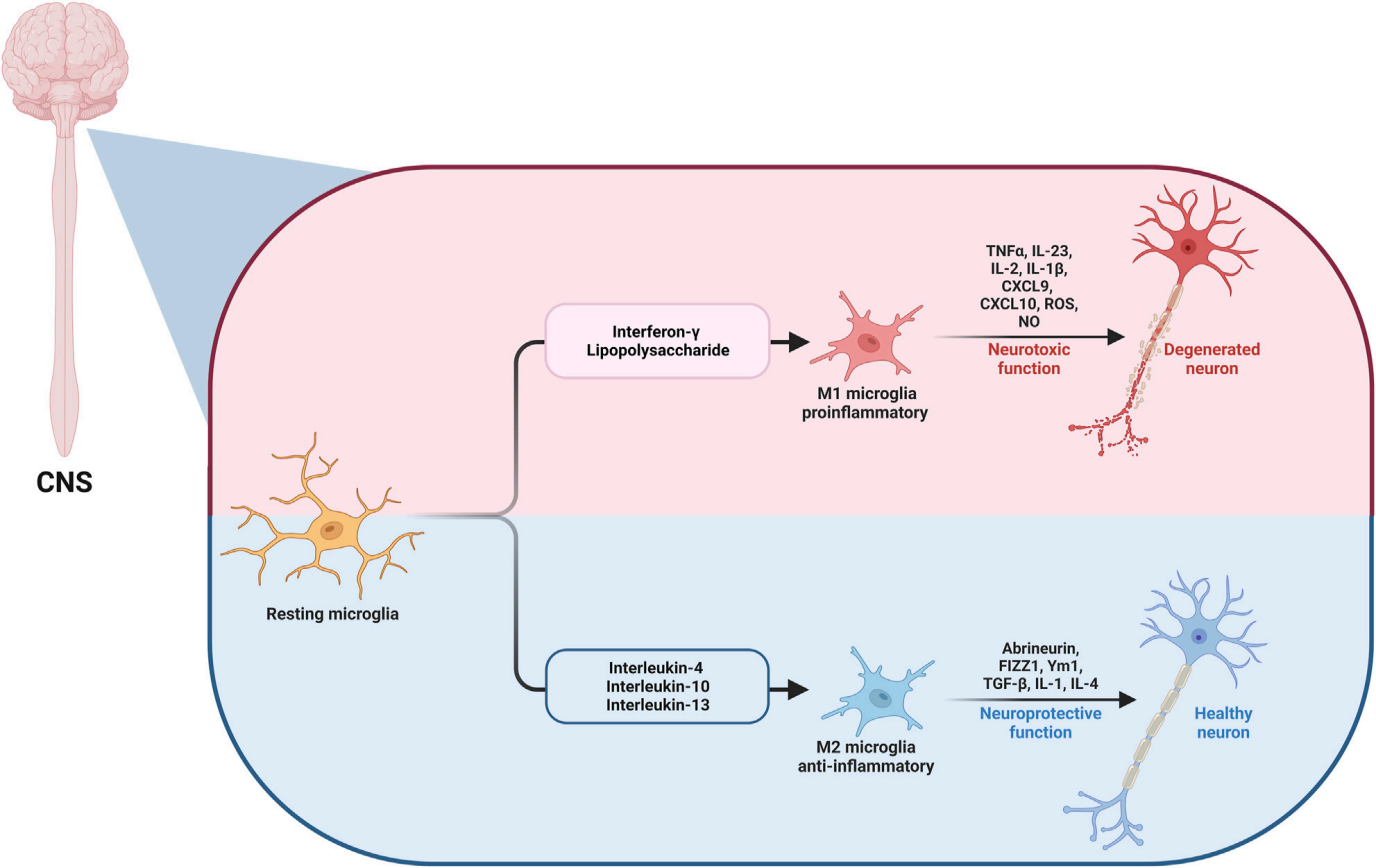
The microglia (M1/M2) dual pathway in CNS.

M1 cells act as the innate immune system’s first line of defense, frequently within the first few hours or days. They use a wide range of immunological receptors to detect harmful stimuli; for example, nucleotide-binding oligomerization domains (NODs), NOD-like receptors, toll-like receptors (TLRs), and multiple scavenger receptors. They are activated by interferon-gamma (IFN-γ) and lipopolysaccharide (LPS). After activation, microglial cells are motivated to release pro-inflammatory factors with neurotoxic effects. Furthermore, IFN-γ stimulates the transcription factor signal transducer and activator of transcription 1 (STAT1) via Janus kinase (JAK)1/JAK2 signaling and stimulates the production of reactive oxygen species (ROS) and nitric oxide (NO) in addition to pro-inflammatory chemotactic factors and cytokines, like tumor necrosis factor-alpha (TNF-α), interleukin-23 (IL-23), IL-2, IL-1β, C-X-C motif chemokine ligand-9 (CXCL9), and CXCL10 (Orihuela et al., 2016). Activation of M1 can also be induced by another pathway through the activation of TLR4 by LPS or damage-associated molecular pattern (DAMP). After that, an “activation complex” is formed, comprising P65, P38, myeloid differentiation factor 88 (Myd88), interferon regulatory factor 3 (IRF3), and nuclear factor kappa B (NFκB), which is a highly conserved transcription factor that controls a variety of crucial physiological processes, including inflammatory reactions, cellular proliferation, and apoptosis. In turn, the complex formed controls the expression of inflammatory mediators from the polarized cell, such as inducible nitric oxide synthase (iNOS), CD16, and CD32, and the major histocompatibility complex-II, CD86, and other cell surface markers (Zhao et al., 2017). On the other hand, M2 microglia are activated by IL-4, IL-10, or IL-13, which motivate microglial cells to release abrineurin, found in inflammatory zone 1, Ym1, and anti-inflammatory cytokines, such as transforming growth factor *ß* (TGF-β), IL-1, and IL-4, which suppress inflammatory responses, encourage repair and regeneration and have neuroprotective effects (Yao and Zu, 2020; Li et al., 2021; Guo et al., 2022). The impact of microglia polarization to M1 or M2 on neurodegeneration and the involved mediators is illustrated in Figure 1.

## 2 Role of non-coding RNAs in microglia (M1/M2) pathway

Non-coding RNAs (ncRNAs) are a diverse class of ncRNA transcripts that do not play any role in protein coding. Nevertheless, it has been proven that they are key factors in many biological processes, including disease progression (Sun and Chen, 2020). Regulatory ncRNAs can be further divided into small non-coding RNAs (sncRNAs), which contain transcripts with fewer than 200 nucleotides (nt), and long non-coding RNAs (lncRNAs), which contain transcripts with more than 200 nt. The three primary types of small ncRNAs are PIWI-interacting RNAs (piRNAs), small-interfering RNA (siRNA), and microRNAs (miRNAs). Certain ncRNAs have different lengths, such as circular RNAs (circRNAs), enhancer RNAs (eRNAs), and promoter-associated transcripts (PATs) may belong to two classes at the same time (Zhang et al., 2019).

ncRNAs play key roles at the post-transcriptional level in different pathways and diseases, including neurodegenerative disorders (Nhung Nguyen et al., 2022; Salama et al., 2022; Elazazy et al., 2023) Table 1. Numerous studies have been conducted on the dynamic ncRNA alterations caused by microglia-induced neuroinflammation (J. Huang et al., 2023; Li et al., 2020; 2022). In the context of Alzheimer’s disease (AD), miRNA-155 regulates synaptic homeostasis of microglia Aβ internalization and synaptic pruning. Deletion of the microglia-specific miRNA-155 resulted in early onset hyper-excitability, frequent spontaneous seizures, seizure-related mortality, and decreased amyloid-beta (Aβ) pathology. As miRNA-155 deletion changed how the microglia internalized synaptic material, this impacted how the microglia mediated the synaptic pruning (Aloi et al., 2023).

**TABLE 1:** The role of different ncRNAs in neurodegenerative diseases.

ncRNAs	Role	Disease	References
miRNA-155	Deletion of miRNA-155 resulted in early onset hyperexcitability, frequent spontaneous seizures, seizure-related mortality, and decreases amyloid-β pathology	Alzheimer’s disease	Aloi et al. (2023)
MALAT1	Inhibits microglial autophagy and inflammatory responses to promote dopaminergic neuronal apoptosis by acting as miR-23b-3p sponge	Parkinson’s disease	Geng et al. (2023)
miRNA-124	Polarization of microglia from M1 to M2, which significantly reduced the neuroinflammation	Neurodegenerative diseases	Huang et al. (2023)
circHIPK3	Increasing neuroinflammation via sponging miRNA-124 and enhanced the release of IL-6, IL-1β, and TNF-α	Parkinson’s disease	Zhang et al. (2022)

Metastatic-associated lung adenocarcinoma transcript 1 (MALAT1), also known as the nuclear-enriched abundant transcript 2, is a lncRNA that is a crucial factor in the pathogenesis of Parkinson’s disease (PD) (Abrishamdar et al., 2022). PD onset and prognosis were correlated with MALAT1-relevant single nucleotide polymorphisms (SNPs), and MALAT1 contributed to increasing the neuronal inflammation of the pathogenesis of PD (Yang, 2021). MALAT1 is overexpressed due to participation in activating inflammatory vesicles in microglia (Geng et al., 2023). In PD models, MALAT1 induces apoptosis of dopamine neurons via sponging miRNA-124 (Liu et al., 2017) and acts as a miR-23b-3p sponge that inhibits microglial autophagy and inflammatory responses to promote dopaminergic neuronal apoptosis. The potential mechanism is that the miR-23b-3p/α-synuclein molecular axis is regulated to promote dopaminergic neuronal cell apoptosis by affecting the endocytosis and intercellular communication of the *a*-synuclein (α-syn) nucleoprotein. As a result, microglia may exhibit impaired autophagy and inflammatory reactions. Consequently, this provides a new treatment target for PD (Geng et al., 2023). Due to its effects on dopaminergic neuron apoptosis, lncRNA MALAT1 may be a new treatment target in PD.

In the CNS, miRNA-124 is highly expressed and perfectly conserved (Wohl and Reh, 2016; Chen et al., 2023). It plays an important role in a variety of neurodegenerative diseases, as well as memory development. Huang et al. found that miRNA-124 polarization of microglia from M1 to M2 significantly reduced the neuroinflammation generated by d-galactose. This was also shown by the presence of iNOS, arginase-1 (Arg-1), and ionized calcium-binding adapter molecule 1 (Iba-1), which also upregulated the anti-inflammatory mediators IL-4 and IL-10 and downregulated the inflammatory mediators TNF-α and IL-1β (J. Huang et al., 2023). Another study focused on miRNA-124 discovered that human serum and cerebrospinal fluid (CSF) both showed significantly higher levels of circHIPK3 expression in PD compared to controls, but miRNA-124 expression was markedly decreased. In BV2 cells (a type of microglial cells), the overexpression of circHIPK3 enhanced the release of IL-6, IL-1β, and TNF-α. Following the expression of circHIPK3, the microglia markers CD11b and Iba-1 protein expressions and pyroptosis-related factors, nucleotide-binding domain, leucine-rich–containing family, pyrin domain–containing-3 (NLRP3), caspase-1, and apoptosis-associated speck-like protein containing a caspase recruitment domain (ASC) were elevated. When miRNA-124 was added, all these effects were reversed. This is due to circHIPK3 increasing neuroinflammation via sponging miRNA-124 and controlling the miRNA-124-mediated STAT3/NACHT, LRR, and PYD domains-containing protein 3 pathway (Zhang et al., 2022). Nutraceuticals may play an important role in the treatment of different diseases by targeting ncRNAs (El-Shehawy et al., 2023; Jiang et al., 2023; Zhang et al., 2023). However, no studies were found that demonstrate an effect of nutraceuticals on the microglia pathway acting via the ncRNAs pathways.

## 3 Neurodegenerative diseases involving microglia (M1/M2) pathway

Neurodegenerative disorders such as AD, PD, Huntington’s disease (HD), amyotrophic lateral sclerosis, and multiple sclerosis (MS) are distinguished by neurodegeneration in particular parts of the CNS and share very similar pathophysiological processes (Song and Suk, 2017). Microglia-induced neuroinflammation has become widely recognized as a dualistic phenomenon in the field of neurodegenerative disorders, comprising both negative and positive effects on neuronal functioning and the surrounding environment (Tang and Le, 2016).

In various neurodegenerative disorders, neuroinflammation caused by microglia, macrophages that are found in the brain, is a prevalent hallmark, and various inflammatory mediators generated by M1 microglia have a role in the development of neurodegeneration and myelin damage in these diseases. Nevertheless, M2 microglia activation is required for tissue maintenance and repair (Jha et al., 2016; Song and Suk, 2017). Multiple studies have confirmed that natural products can both prevent and treat neurodegenerative diseases by influencing the polarization of microglia towards M1/M2 phenotypes. These natural compounds can potentially inhibit or reduce the inflammatory toxicity of M1 microglia. Furthermore, they can help repair and regenerate damaged neurons, axons, or myelin by improving the release of neurotrophic factors or cytokines from M2 microglia (Jin et al., 2019).

### 3.1 Alzheimer’s disease

#### 3.1.1 Role of microglia in Alzheimer’s disease

Alzheimer’s disease is the prevailing type of dementia, associated with damaged locomotor ability, thinking, judgment ability, increasing memory loss, and cognitive decline (Fu et al., 2016). It is distinguished by the abnormal presence of Aβ-containing plaques extracellularly and the creation of neurofibrillary tangles inside the cell composed of hyperphosphorylated tau protein (Prince et al., 2016; Baufeld et al., 2017).

Postmortem analysis revealed an overwhelming number of “plaques” and “tangles” as distinguishing signs of AD. These senile plaques are accumulations of fibrils and aggregations of Aβ located outside of cells caused by abnormal proteolytic degradation of the amyloid precursor protein (APP), which is enhanced by presenilin-1 (PS1) (Citron et al., 1992; Mattson, 2004). *In vivo*, amyloid plaques can attract and stimulate microglia cells (Meyer-Luehmann et al., 2008), while *in vitro* studies have demonstrated that Aβ peptides can stimulate primary microglia activation and promote NO generation. However, this microglia activation can result in the adoption of many phenotypes, which is further increased by the presence of amyloid fibrils and extracellular Aβ peptides (Walker et al., 2006; Maezawa et al., 2011).

Furthermore, neuroinflammation has an important role in the etiology of AD (Baufeld et al., 2017). In the CSF of mild memory impairment patients who developed AD, researchers discovered higher TNF-α (a pro-inflammatory cytokine) and lower TNF-β (anti-inflammatory cytokine) levels compared to controls who had not experienced AD (Tarkowski et al., 2003). The neuroinflammatory response is demonstrated by modifications to the structure of microglia and astrocytes in proximity to senile plaques (Glass et al., 2010). Both astrocytes and microglia interact with Aβ, and interruptions in their metabolism and functioning can result in Aβ depositions (Yan et al., 2013; Hickman et al., 2018). As a result, Aβ uses TLRs to stimulate astrocytes and microglia, promoting neurodegeneration by causing the production of neuroinflammatory mediators (Glass et al., 2010).

Microglia also can respond to potentially damaging stimuli such as misfolded Aβ proteins (Sarlus and Heneka, 2017). M2 microglia, which are mainly accountable for up-taking and eliminating insoluble fibrillar Aβ deposits, perform a protective function in the brain. Microglia can break down Aβ by producing enzymes such as insulin-degrading enzymes (Heneka, 2017), hence minimizing AD incidence (Hansen et al., 2018).

At the outset of Aβ pathology, the microglia that encircle the Aβ plaques are typical of the neuroprotective phenotype, identified as Ym1. Thus, Microglia perform an important role in reducing the accumulation of potentially neurotoxic Aβ aggregates while also protecting neurons from localized toxicity (Condello et al., 2015). However, an age-dependent rise of both the size and amount of Aβ plaques in AD may represent a reduction in microglial phagocytic abilities (Mawuenyega et al., 2010), and this neuroprotective phenotype eventually changes to the neurologically harmful pro-inflammatory one at the final stages of the disease (Tang and Le, 2016).

Pro-inflammatory cytokines reduce microglia phagocytic activity and additionally are likely to shift microglia into pro-inflammatory phenotypes. Consequently, pro-inflammatory microglia promote tau phosphorylation (Lee et al., 2010). Furthermore, Microglia release neurotoxic cytokines that directly harm neurons or activate neurotoxic astrocytes (Hansen et al., 2018). It was also revealed that synaptic loss and aberrant tau phosphorylation in AD are caused by the dysregulation of Wnt pathways and that Wnt signaling regulates microglial inflammation (Yang and Zhang, 2020).

The dynamic nature of microglial activation involves constant transitions between different phenotypes. In a study conducted by Fan et al., it was suggested that microglial activation in AD may exhibit two distinct peaks (Fan et al., 2017). The first peak, occurring in the preclinical stage, is characterized by an anti-inflammatory response. The second peak, observed in the clinical stage as the disease advances and Aβ clearance mechanisms fail, demonstrates a pro-inflammatory reaction. These findings align with microglia’s dual role in AD pathogenesis.

#### 3.1.2 Nutraceuticals that influence microglial activation in Alzheimer’s disease

**FIGURE 2:**
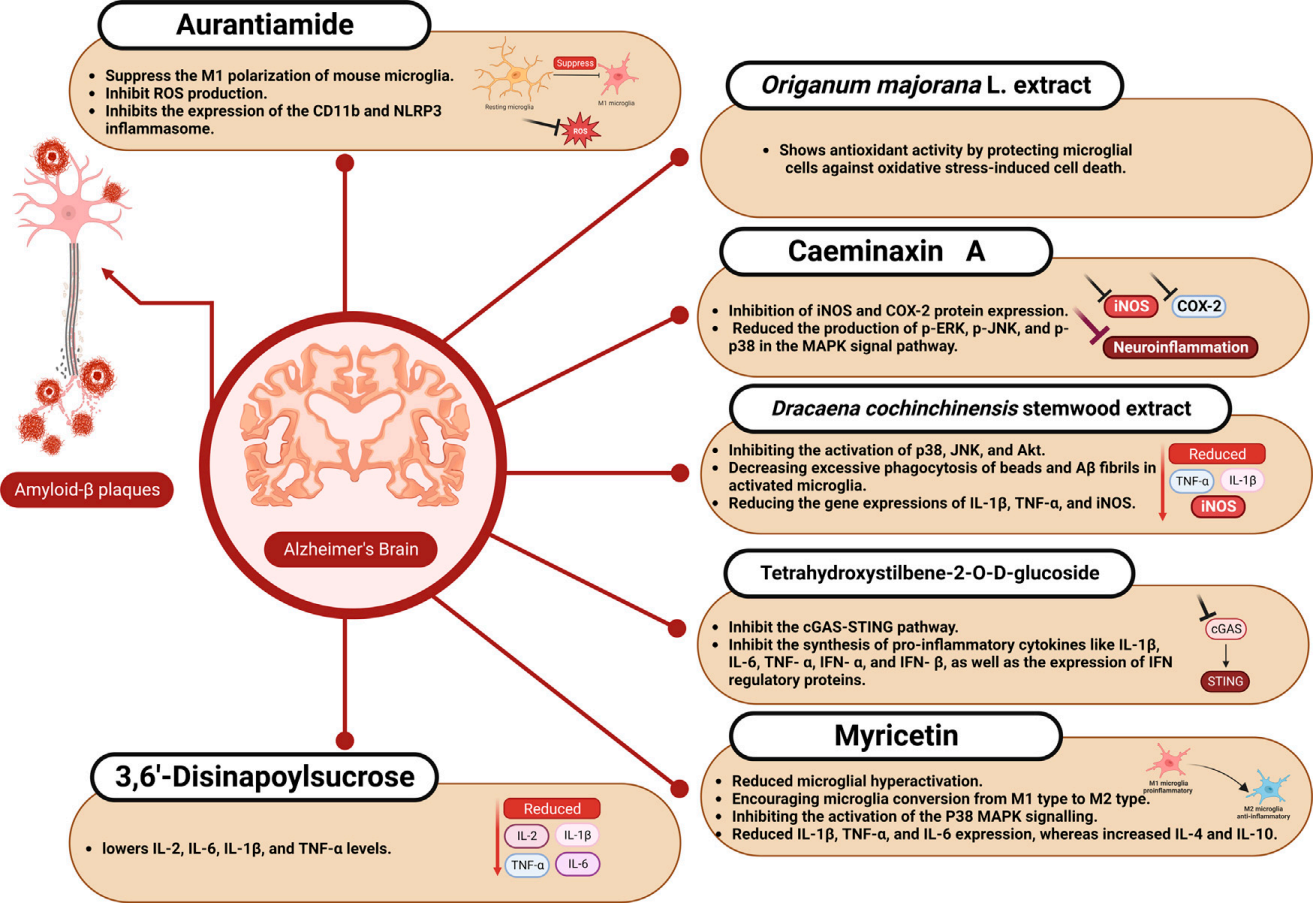
The effect of aurantiamide, myricetin, and other nutraceuticals on microglial activation in Alzheimer’s disease.

**FIGURE 3:**
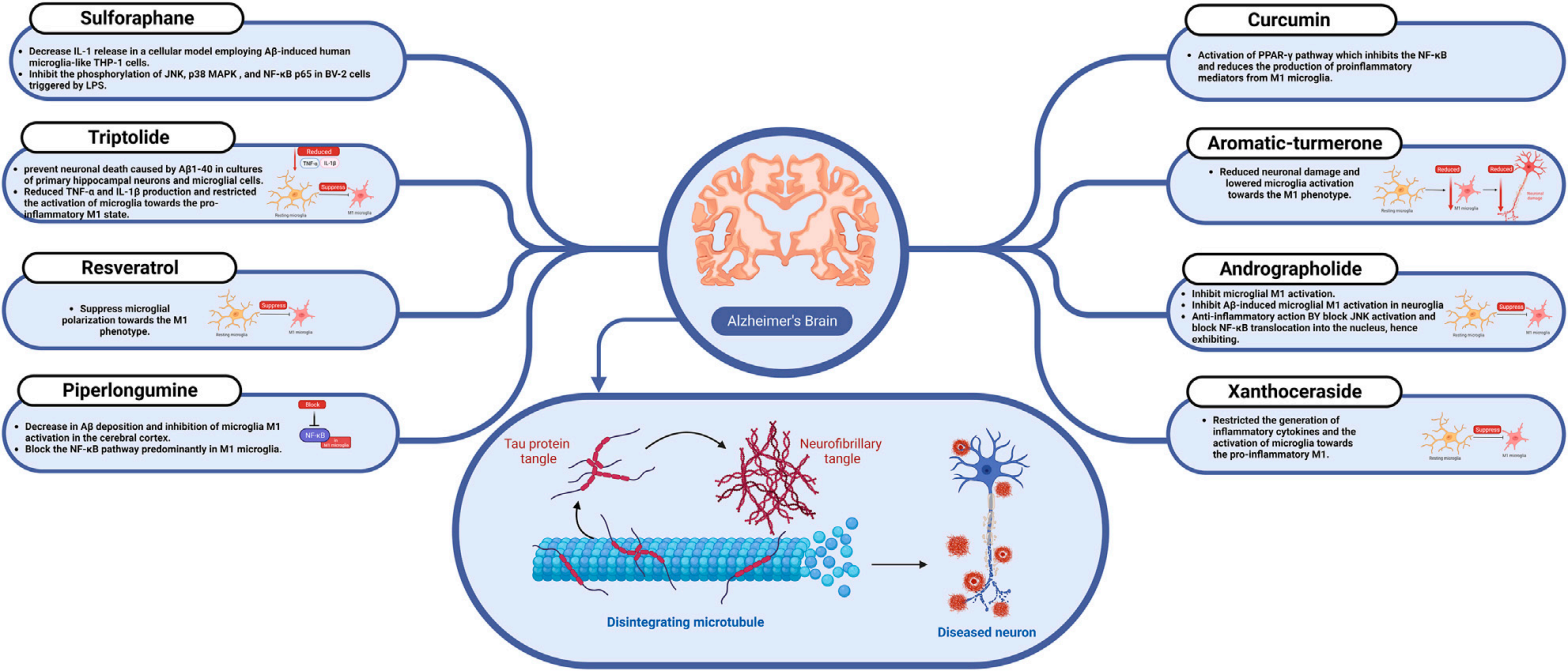
The effect of sulforaphane, resveratrol, curcumin, and other nutraceuticals on microglial activation in Alzheimer’s disease.

The impact of different natural products and nutraceuticals on AD through regulation of M1/M2 microglia polarization is presented in Table 2 and Figures 2, 3.

**TABLE 2:** The effects of different nutraceuticals on microglia in Alzheimer’s disease related models.

Compound/Extract	Model	Concentration/dose	Biological effects	References
*Origanum majorana* L. extract	*In vitro* study using BV-2 microglia cells and	Cells were pre-treated with OM extract (25, 50, 100, 200 μg/mL) or rosmarinic acid (5, 10, 20 μg/mL) for 24 h	Shows antioxidant activity by protecting microglial cells against oxidative stress-induced cell death	Wagdy et al. (2023)
	*In vivo* LPS-injected Swiss albino mouse model	Mice were treated with OM extract at the dose of 100 mg/kg/day, i.p. for 12 days	Reduction of neurodegeneration and neuroinflammation in mice brains	
Caeminaxin A	BV-2 microglia cells stimulated by LPS	3, 10, and 30 μM for 24 h	• Inhibition of iNOS and COX-2 protein expression at 30 μM	Lu et al. (2023)
			• Reduced the production of p-ERK, p-JNK, and p-p38 in the MAPK signal pathway	
*Dracaena cochinchinensis* stemwood extract	BV-2 microglia cells and RAW264.7 cells stimulated by LPS	Cultures pretreated with DCS_EtOH90_ (0.5–10 μg/mL)	• Decreasing excessive phagocytosis of beads and Aβ fibrils	Ospondpant et al. (2023)
		DCS_EtOH50_ (0.5–10 μg/mL)	• Reducing the gene expression of IL-1β, TNF-α, and iNOS.	
		DCS_water_ (5–100 μg/mL) for 4 h before exposure to 100 ng/mL of LPS for an additional 20 h	• Enhancing the expression of the anti-inflammatory Arg-1 in both BV-2 microglia and RAW264.7 macrophages	
			• Inhibiting the phosphorylation and activation of p38, JNK, and Akt	
Tetrahydroxystilbene-2-O-D-glucoside	APP/PS1 mice	40 and 80 mg/kg/day, p.o., for 5 months	• Decreased cGAS, STING and decreased the expression of NLRP3	Gao et al. (2023a)
	BV-2 microglia cells stimulated by LPS/IFN-γ	Cells were treated with 25, 50, and 100 μM for 20 h	• Inhibit the cGAS-STING pathway	
			• Inhibit the synthesis of pro-inflammatory cytokines like IL-1β, IL-6, TNF- α, IFN- α, and IFN- β, as well as the expression of IFN regulatory proteins	
Myricetin	BV-2 microglia cells stimulated with Aβ_25–35_	50 μmol/L for 48 h	• Inhibiting the activation of the P38 MAPK signaling that prevents	Liu et al. (2023)
			• Hyperactivation of microglia	
			• Conversion from the M2 to the M1 microglia type	
			• NLRP3 activation	
	3 × Tg-AD mice	20 mg/kg/day for 3 weeks	• Reduced neuroinflammation through reduced IL-1β, TNF-α, and IL-6 expression, whereas increased IL-4 and IL-10	
			• Reduced microglial hyperactivation	
			• Encouraging microglia conversion from M1 type to M2 type	
Aurantiamide	BV-2 microglia cells stimulated by LPS/IFN-γ	Cells were pretreated with 10 μM and 20 μM for 6 h	• Suppress the M1 polarization of mouse microglia	Shen et al. (2023)
			• Inhibit ROS production	
			• Inhibits the expression of the CD11b and NLRP3 inflammasome	
			• Decreases the expression of the inflammatory cytokines IL-6, TNF-α, and IL-1β	
3,6′-Disinapoylsucrose	AD model mice by administering APPswe695 lentivirus	1, 3, and 9 mg/kg	• lowers IL-2, IL-6, IL-1β, and TNF-α levels	Wang et al. (2023)
Curcumin	Animal (APP/PS1 mice)	150 mg/kg/day, i.p., for 4 weeks	Specific targeting of PPAR-γ inhibits the NFκB and reduces the production of pro-inflammatory mediators from M1 microglia	Liu et al. (2016)
Aromatic-turmerone	*In vitro* study using BV-2 cells isolated from mice	5, 10 and 20 μM for 1 h	Inhibition of JNK, p38-MAPK pathways, and reducing the production of pro-inflammatory cytokines, chemokines, and ROS.	Park et al. (2012a)
	*In vitro* study using LPS-stimulated BV-2 cells	5, 10 and 20 μM for 1 h	Inhibition of the STAT, NFκB, and MAPK pathways	Park et al. (2012b)
Resveratrol	*In vitro* study using LPS-stimulated murine RAW 264.7 and microglial BV-2 cells	Cells were pre-treated with 50 μM resveratrol for 30 min before LPS stimulation	Reduction of LPS or Aβ-mediated microglial M1 stimulation by blocking the TLR4/NFκB/STATs signaling pathway	Capiralla et al. (2012)
	*In vivo* study using APP/PS1 transgenic mice	AIN-93G diet supplemented with 0.35% resveratrol for 15 weeks		
	Double-blind phase II clinical study in individuals with mild to severe AD	500 mg/day, p.o., with dose escalation by 500-mg increments every 13 weeks, for 52 weeks	Efficacious, safe, and tolerable in individuals with mild to severe AD. Significant improvements in spatial learning and memory impairments	Turner et al. (2015)
Andrographolide	*In vivo* (APP/PS1 mice and elderly degus)	2 mg/kg, i.p., 3 times per week	Substantial alleviation of pathological and behavioral abnormalities associated with AD in both APP/PS1 mice and elderly degus	Serrano et al. (2014), Rivera et al. (2016)
	*In vitro* study using BV-2 and Neuro-2A cells culture	0.5–10 μM 1 h before Aβ_1-42_ treatment	• Inhibition of Aβ-induced microglial M1 activation in neuroglia cultures	Yang et al. (2017)
			• Blockage of JNK activation and NFκB translocation into the nucleus	
Sulforaphane	*In vitro* study using human microglia-like THP-1 cells	5–50 μM for 30 min before Aβ_1–42_ treatment	• Decreased IL-1 release in a cellular model employing Aβ-induced human microglia-like THP-1 cells	An et al. (2016)
			• Inhibition of STAT1 phosphorylation and activation of the NLRP3 inflammasome. Activation of the Nrf2/HO-1 pathway	
	*In vitro* (BV-2 cells derived from C57/BL6 murine)	5–15 μM for 12 h	• Inhibition of the phosphorylation of JNK, p38 MAPK, and NFκB p65	Qin et al. (2018)
			• Reduction of microglia-mediated necroptosis and apoptosis through the suppression of pro-inflammatory responses	
Triptolide	*In vitro* using primary hippocampal neurons and microglial cells	5–25 μg/mL for 24 h	Prevention of neuronal death caused by Aβ1-40 in primary hippocampal neurons and microglial cells	Nie et al. (2012)
	*In vivo* using an AD mouse model (APP/PS1 mice)	20 μg/kg/day, i.p., for 15 weeks	Reduction of TNF-α and IL-1β production, restriction of microglial activation towards the pro-inflammatory M1 state, inhibition of the phosphorylation of the MAPKs signaling pathway	Cui et al. (2016)
Xanthoceraside	*In vitro* murine microglial cell line N9	0, 0.001, 0.01, and 0.1 μM for 16 h	Inhibition of the release of NO and pro-inflammatory cytokines: IL-1β and TNF-α	Qi et al. (2013)
	*In vivo* using mice injected with Aβ1-42	0.02, 0.08, and 0.32 mg/kg/day, p.o., for 9 days	Inhibition of the translocation of NFκB p50 and p65 into the nucleus and suppressing MAPK phosphorylation	Qi et al. (2018)
Piperlongumine	*In vivo* study using Tg-APPswe/PS1dE9 transgenic mice (APP/PS1)	50 mg/kg/day, p.o., for 10 weeks	Decreased Aβ deposition and inhibition of microglia M1 activation in the cerebral cortex	Go et al. (2018a)
	*In vivo* study using female C57BL/6 J mice	50 mg/kg/day, p.o., for 8 weeks	Blocking the NFκB pathway in M1 microglia	Go et al. (2018b)

##### 3.1.2.1 Origanum majorana L.

*Origanum majorana* L. is an aromatic plant used to treat various diseases in folk medicine, including intestinal antispasmodic, intestinal hypertension, allergies, respiratory infections, diabetes, and stomach pain (Bouyahya et al., 2021). *Origanum majorana* is a plant rich in phenolic compounds like rosmarinic acid and its derivatives. *Origanum majorana* extract and rosmarinic acid strongly protected microglial cells from oxidative stress-induced cell death by the antioxidant activity of rosmarinic acid. *Origanum majorana* additionally protected mice from the alterations in recognition and spatial memory induced by LPS and reduced expression of glial fibrillary acidic protein and cyclooxygenase-2 (COX-2) in mouse brain tissue (Wagdy et al., 2023).

##### 3.1.2.2 Caesalpinia dinax

*Caesalpinia minax* Hance is a species of plant that belongs to the Fabaceae family and spreads throughout Southeast Asia’s tropical and subtropical regions. This plant’s seeds, known in China as “Ku-shi-lian,” have long been used in traditional medicine to treat fever, diarrhea, and the common cold (Jing et al., 2019; Ruan et al., 2019). Twenty cassane diterpenoids were identified in the leaves of Caesalpinia minax, including two unique ones (caeminaxins A and B). To evaluate the anti-neuritis activity of these 20 compounds, Lu et al. measured the amount of NO production of mouse microglia BV-2 cells stimulated by LPS as neuro-inflammatory diseases are characterized by an increase in NO production. The most potent inhibitory effect was produced by caeminaxin A. Different concentrations of this compound (3, 10, and 30 μM) were used to treat BV-2 cells stimulated by LPS. Caeminaxin A at 30 μM significantly inhibited iNOS and COX-2 protein expression. Additionally, Caeminaxin A reduced the production of p-ERK, p-JNK, and p-p38 in the mitogen-activated protein kinase (MAPK) signal pathway, which suppressed neuroinflammation (Lu et al., 2023).

##### 3.1.2.3 Dracaena cochinchinensis

*Dracaena cochinchinensis* is a tropical forest plant belonging to the family Asparagaceae (Wu et al., 2019). *Dracaena cochinchinensis* stemwood is used in traditional Chinese medicine to treat blood stasis, severe injuries, and pain (Fan et al., 2014; Ospondpant et al., 2022). In LPS-activated microglia, *D. cochinchinensis* stemwood extract reduced the neuroinflammatory response by reducing pro-inflammatory factors and inhibiting the activation of p38, JNK, and Akt and by reducing the gene expressions of IL-1β, TNF-α, and iNOS. Additionally, *D. cochinchinensis* stemwood extract could decrease excessive phagocytosis of beads and Aβ fibrils in activated microglia (Ospondpant et al., 2023). *Dracaena cochinchinensis* stemwood extract can also prevent the formation of Aβ fibrils, protecting against Aβ-mediated cell damage and stimulating neural differentiation (Ospondpant et al., 2022).

##### 3.1.2.4 Polygonum multiflorum

*Polygonum multiflorum* is traditional Chinese herbal medicine used for centuries as a treatment for a wide range of conditions, including dizziness, liver disease, graying of the hair, and constipation (Xue et al., 2020; Guo et al., 2023). Chinese knotweed, Fo-Ti, Shou Wu Pian, and He Shou Pian are other names for *P. multiflorum* (Gumber and Barmota, 2023). The main active compound from Polygonum multiflorum is tetrahydroxystilbene-2-O-D-glucoside (TSG, C_20_H_22_O_9_) (Wang et al., 2022). TSG caused a decrease in cyclic GMP-AMP synthase (cGAS), a decrease in the immune response that was induced by the stimulator of interferon genes (STING), and decreased the expression of NLRP3 inflammasome by inhibition of the activation of the cGAS-STING pathway in APP/PS1 mice. Additionally, cell culture using LPS and IFN-γ to activate microglia indicated that TSG reversed the polarization status of M1-type microglia to restore quiescence and inhibit cGAS-STING pathway as active microglia showed higher cGAS-STING levels. TSG also reduced the inflammatory response induced by LPS/IFN-γ in BV2 cells by inhibiting the synthesis of pro-inflammatory cytokines such as IL-1β, IL-6, TNF- α, IFN- α, and IFN- β, as well as the expression of IFN regulatory proteins like IFIT1 and IRF7 (Gao et al., 2023a).

##### 3.1.2.5 Myricetin

Myricetin is a flavonoid compound in various natural plants (Song et al., 2021). It can be found in foods, including fruits, vegetables, tea, and wine. The richest sources of myricetin are the families *Myricaceae, Polygonaceae, Primulaceae, Pinaceae,* and *Anacardiaceae.* Previous studies have demonstrated that myricetin has various pharmacological effects, including its anticancer, anti-diabetic, anti-obesity, cardiovascular, anti-osteoporosis, anti-inflammatory, and hepatoprotective (Imran et al., 2021). Myricetin has neuroprotective action, which has been demonstrated in preclinical studies on amyotrophic lateral sclerosis, PD, AD, and HD (Taheri et al., 2020). According to the findings of *in vitro* AD model (BV-2 microglia cells stimulated with Aβ_25–35_), myricetin prevented the hyperactivation of microglia and the conversion from the M2 to the M1 type and inhibited NLRP3 activation by inhibiting the activation of the P38 MAPK signaling pathway. In 3 × Tg-AD mice, myricetin reduced microglia hyperactivation, encouraged microglia conversion from M1 type to M2 type, and effectively reduced neuroinflammation through reduced IL-1β, TNF-α, and IL-6 expression, whereas increased IL-4 and IL-10. In addition, it could significantly enhance memory loss, spatial learning capacity, Aβ plaque formation, and neuronal and synaptic damage (Liu et al., 2023).

##### 3.1.2.6 Aurantiamide

Aurantiamide is a natural product found in various plants, such as *Portulaca oleracea* L. and *Zanthoxylum dissitum*. It shows a variety of biological activities like anti-HIV, anti-inflammatory, antibacterial, and antioxidant effects (Suhas and Channe Gowda, 2012; Chen et al., 2016). Aurantiamide was found to decrease central neuroinflammation, inhibit ROS production, suppress the M1 polarization of mouse microglia, and enhance cognitive performance in mice. Additionally, Aurantiamide inhibits the expression of the CD11b and NLRP3 inflammasome and also decreases the expression of the inflammatory cytokines IL-6, TNF-α, and IL-1β (Shen et al., 2023).

##### 3.1.2.7 3,6′-Disinapoylsucrose

3,6′-Disinapoylsucrose is an oligosaccharide ester bioactive compound obtained from *Polygalae Radix* that significantly reduces depression (Zhang et al., 2022). In AD model mice, 3,6′-Disinapoylsucrose can greatly reduce neuroinflammation, problems with spatial learning, and memory issues. 3,6′-Disinapoylsucrose enhances cognitive function, lowers IL-2, IL-6, IL-1β, and TNF-α levels, reduces NFκB p65 expression, and reduces Aβ deposition and nerve cell injury. 3,6′-Disinapoylsucrose can control the hippocampus’s tyrosine kinase B/brain-derived neurotrophic factor signaling (Wang et al., 2023).

##### 3.1.2.8 Curcumin

Curcumin is an important polyphenolic component found in the rhizome of Curcuma longa (*Zingiberaceae*). It has a rich history of traditional use as a food and herbal medicine in Asia and has emerged as a highly promising phytochemical candidate for treating AD owing to its notable anti-inflammatory and immunomodulatory properties (Ruby et al., 1995). Administering curcumin to APP/PS1 mice at a dose of 150 mg/kg through intraperitoneal injection for 4 weeks significantly improved spatial learning and memory impairments. Its effects were notable for efficiently inhibiting the NFκB signaling pathway and lowering the production of pro-inflammatory mediators from M1 microglia. It specifically targeted peroxisome proliferator-activated receptor gamma (PPAR-γ) to obtain these positive results (Liu et al., 2016).

Several challenges arise from the pharmacokinetic properties of curcumin, including rapid degradation, low bioavailability, and limited solubility in body fluids. Various innovative drug delivery systems for curcumin have been developed to address these limitations. One such system is the solid-lipid curcumin particle, which has demonstrated remarkable enhancements in anti-amyloid, anti-inflammatory, and neuroprotective effects compared to natural curcumin (Maiti et al., 2018).

##### 3.1.2.9 Aromatic-turmerone

Aromatic-turmerone, a prominent phytoconstituent found in Curcuma longa essential oil, has a comparable chemical structure and potential bioactivities to curcumin and 6-shogaol derived from ginger. In an *in vitro* study, the aromatic-turmerone was shown to decrease the synthesis of pro-inflammatory chemicals in BV-2 cells, derived from C57/BL6 murine, which is a well-characterized and extensively employed model system for microglia, triggered by LPS via inhibiting the STATs and mitogen-activated protein kinase pathways (Park et al., 2012b). Another *in vitro* study found that administrating aromatic-turmerone reduced neuronal damage and lowered microglia activation towards the M1 phenotype. These data support the anti-inflammatory and neuroprotective properties of aromatic-turmerone. These positive benefits were connected to avoiding neuronal injury by limiting microglial M1 activation and decreasing inflammatory cytokine production (Park et al., 2012a).

##### 3.1.2.10 Resveratrol

Many different plants produce resveratrol, a natural phenol, and stilbenoid, as a defense mechanism against damage or pathogen attacks. It is plentiful in grape skin and blueberries and has significant anti-inflammatory benefits. Numerous studies have shown that it can suppress microglial polarization towards the M1 phenotype and improve cognitive deficits in cellular and animal models of dementia. In addition, Resveratrol reduced LPS or Aβ-mediated microglial M1 stimulation by blocking the TLR4/NFκB/STATs signaling pathway (Capiralla et al., 2012). Furthermore, resveratrol was shown to be efficacious, safe, and tolerable in individuals with mild to severe AD in a double-blind phase II clinical study (Turner et al., 2015).

##### 3.1.2.11 Andrographolide

Andrographolide is a labdane diterpenoid present throughout the whole *Andrographis paniculata* plant. It has attracted interest due to its powerful anti-inflammatory characteristics and potential for treating AD. Andrographolide, taken intraperitoneally at a dose of 2.0 mg/kg, substantially alleviated pathological and behavioral abnormalities, which implies AD in both APP/PS1 mice and elderly degus (56-month-old) as demonstrated in the *in vivo* studies (Serrano et al., 2014; Rivera et al., 2016). Furthermore, *in vitro* investigations have demonstrated that andrographolide can inhibit microglial M1 activation. Andrographolide inhibited Aβ-induced microglial M1 activation in neuroglia cultures when administered at a dose of 5 μM. Andrographolide has been shown in mechanistic studies to block JNK activation and block NFκB translocation into the nucleus, hence exhibiting anti-inflammatory actions (Yang et al., 2017).

##### 3.1.2.12 Sulforaphane

Sulforaphane, an organosulfur isothiocyanate substance, is mainly found in cruciferous vegetables such as broccoli, mustard radish, and cabbage. It is a powerful activator of the nuclear factor erythroid 2–related factor 2/heme oxygenase 1 (Nrf2/HO1) pathway with a wide range of biological and pharmacological actions, including anti-inflammatory and antioxidant properties (Zhang et al., 2017). An *in vitro* study found that sulforaphane at a dose of 5 μM decreased IL-1 release in a cellular model employing Aβ-induced human microglia-like THP-1 cells. This was accomplished via inhibiting STAT1 phosphorylation and activation of the NLRP3 inflammasome. The activation of the Nrf2/HO-1 pathway relates to sulforaphane’s latent mechanism (An et al., 2016). Furthermore, previous *in vitro* research found that sulforaphane at doses ranging from 5 to 15 μM inhibited the phosphorylation of JNK, p38 MAPK, and NFκB p65 in mentioned BV-2 cells, derived from C57/BL6 murine, triggered by LPS. Microglia-mediated necroptosis and apoptosis were reduced due to this indirect suppression of pro-inflammatory responses (Qin et al., 2018).

##### 3.1.2.13 Triptolide

Triptolide, a diterpenoid molecule produced from the plant *Tripterygium wilfordii*, is well-known for its medicinal promise in the treatment of inflammatory and autoimmune conditions such as systemic lupus erythematosus, rheumatoid arthritis, and nephrotic syndrome. Additionally, multiple *in vitro* studies have shown that triptolide at 5 and 25 μg/mL doses successfully prevented neuronal death caused by Aβ1-40 in cultures of primary hippocampal neurons and microglial cells (Nie et al., 2012). Another *in vivo* study using an AD mouse model (APP/PS1 mice) discovered that triptolide (20 g/kg intraperitoneally for 15 weeks) effectively reduced TNF-α and IL-1β production and restricted the activation of microglia towards the pro-inflammatory M1 state. These results were obtained by inhibiting the phosphorylation of the MAPK signaling pathway (Cui et al., 2016).

##### 3.1.2.14 Xanthoceraside

Xanthoceraside is a novel triterpenoid saponin isolated from the husk of *Xanthoceras sorbifolia* that has traditionally been used as an anti-rheumatic treatment (Zhou et al., 2022). Xanthoceraside has a remarkable promise in treating AD caused by inflammation via targeting the NFκB pathway. In a recent *in vivo* investigation, oral administration of xanthoceraside (0.08 or 0.32 mg/kg for 9 days) significantly restricted the generation of inflammatory cytokines and the activation of microglia towards the pro-inflammatory M1 state in the hippocampus of mice injected with Aβ1-42. Blocking the translocation of NFκB p50 and p65 into the nucleus and suppressing the phosphorylation of MAPK signaling pathways is considered the main xanthoceraside inhibitory mechanism (Qi et al., 2018; 2013).

##### 3.1.2.15 Piperlongumine

Piperlongumine, an amide alkaloid produced from the fruit of the long pepper plant (*Piper longum*), which is native to southern India and Southeast Asia, has piqued the interest of pharmacologists due to its possible medicinal qualities (Kumar et al., 2011). Numerous studies have been undertaken employing various animal models to evaluate the effects of piperlongumine on AD-like pathology. Piperlongumine (50 mg/kg/day, i. g, for 2.5 months) was given to APP/PS1 mice in an *in vivo* study, resulting in significant restoration of cognitive function (Go et al., 2018b). This improvement was attributable to a decrease in Aβ deposition and inhibition of microglia M1 activation in the cerebral cortex (Go et al., 2018a). Furthermore, previous studies have proven that piperlongumine has neuroprotective benefits in AD rat models via blocking the NFκB pathway predominantly in M1 microglia (Gu et al., 2018).

### 3.2 Parkinson’s disease

#### 3.2.1 Role of M1/M2 in Parkinson’s disease

Just behind AD, PD ranks as the second most prevalent neurodegenerative disorder. It is distinguished by the slow degeneration of dopamine neurons in the midbrain’s substantia nigra pars compacta (SNpc) and A1 neurotoxic astrocyte activation. Lewy bodies (LBs), which are primarily composed of filamentous *a*-syn, are a distinguishing hallmark of PD. These protein aggregates are a fundamental illness feature and are important in its pathogenesis. Additionally, there is an excessive proliferation of reactive microglia, a type of immune cell in the brain (Eriksen et al., 2005; Tang et al., 2013; Song and Suk, 2017). Through functional alterations, astrocytes play key roles in PD pathogenesis (González-Reyes et al., 2017). Dopamine regulates Ca^2+^ signals in astrocytes (Vaarmann et al., 2010). Dopamine neuron loss may alter astrocyte Ca^2+^ homeostasis, and Ca^2+^ imbalance may result in the generation of toxic compounds and cell death in PD (Zaichick et al., 2017). Furthermore, emerging data from multiple studies suggest that glia maturation factor (GMF) produced from astrocytes stimulates the NFκB signaling pathway and consequent granulocyte macrophage-colony stimulating factor (GM-CSF) release. Increased levels of GM-CSF have been implicated in microglia activation and subsequent generation of inflammatory molecules such as IL-1β, TNF-α, and macrophage inflammatory proteins-1 beta (MIP-1β) (Zaheer et al., 2007; Fan et al., 2018).

The presence of *a*-syn in dopaminergic cells has been associated with increased amounts of ROS. This implies that *a*-syn may contribute to oxidative damage by influencing mitochondrial activity. Overexpression of mutant *a*-syn has been demonstrated to make dopaminergic neurons more susceptible to mitochondrial toxins such as 6-hydroxydopamine (6-OHDA) and mitochondrial processing peptidases 1-methyl-4-phenylpyridinium ion (MPP+), leading to elevated protein carbonylation and lipid peroxidation. These results demonstrate that *a*-syn may regulate oxidative stress and mitochondrial dysfunction in dopaminergic cells (Chinta and Andersen, 2008).

Microglia-mediated neuroinflammation has a complicated function in PD since it can have both neuroprotective and neurotoxic effects. Microglia are triggered in the early stages of PD by factors such as *a*-syn, infections, or environmental pollutants. At this stage, microglia are generally static and have little relationship with the severity of clinical symptoms (Tang and Le, 2016). During this initial stage, microglia secrete anti-inflammatory cytokines to decrease the inflammatory response and promote tissue healing and repair (Du et al., 2018). This activation is critical for immunological defense and survival of neurons (Tang et al., 2013). However, when PD advances, persistent microglial activation becomes harmful. Prolonged stimulation can exacerbate motor impairments and cause extensive neuronal damage in neighboring areas (Song and Suk, 2017). Moreover, aggregated *a*-syn can directly motivate microglia to adopt a pro-inflammatory M1 phenotype. This aggravates motor impairments and severely damages adjacent neurons. Understanding the dynamic nature of microglial activation and its role in the etiology of PD is critical for developing treatment techniques to control neuroinflammation and offer neuroprotection (Tang and Le, 2016; Song and Suk, 2017; Du et al., 2018).

#### 3.2.2 Nutraceuticals that influence microglial activation in Parkinson’s disease

**FIGURE 4:**
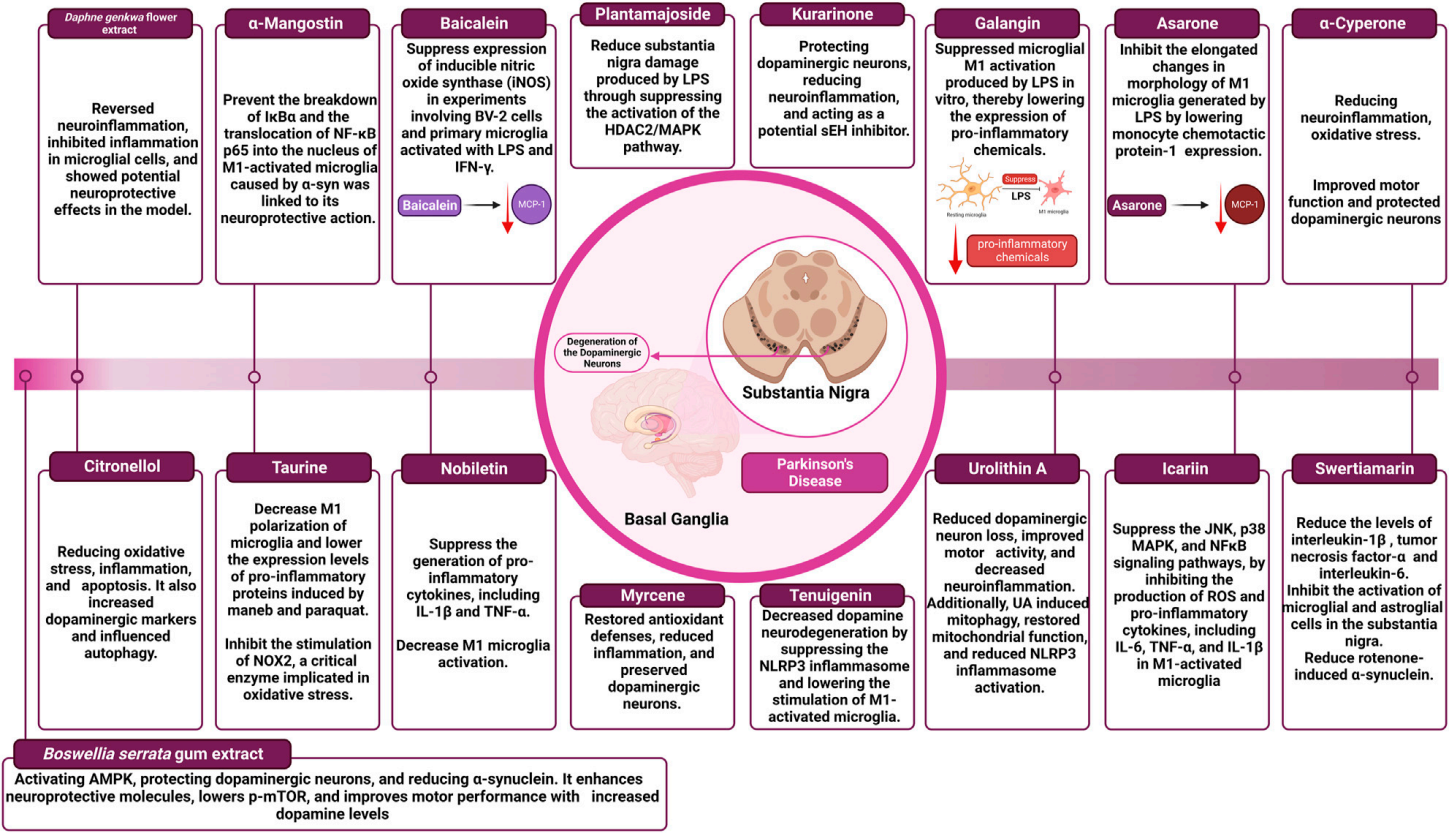
The effect of different nutraceuticals on microglial activation in Parkinson’s disease.

The impact of different natural products and nutraceuticals on PD through regulation of M1/M2 microglia polarization is presented in Table 3 and Figure 4.

**TABLE 3:** The effects of different nutraceuticals on microglia in Parkinson’s disease related models.

Compound	Model	Concentration/dose	Biological effects	References
α-Cyperone	*In vivo* rat model by LPS	10 mg/kg/day for 4 weeks	Reduction of neuroinflammation, oxidative stress, and enhancing antioxidant enzymes. It improved motor function and protected dopaminergic neurons	Huang et al. (2023)
Myrcene	*In vivo* mouse model by ROT	50 mg/kg, p.o., 30 min before ROT injection at the dose of 2.5 mg/kg, i.p. Treatments were carried out 5 days a week, for 28 days	Restored antioxidant defenses, reduced inflammation, and preserved dopaminergic neurons. It indicates a potential neuroprotective role for myrcene against the toxic effects of ROT.	Azimullah et al. (2023)
Daphne genkwa flower extract (GFE)	*In vivo* model by LPS injection in C57BL/6 J mice	50, 100, and 200 mg/kg/day, p.o. for 3 days	GFE reversed neuroinflammation, inhibited inflammation in microglial cells, and showed potential neuroprotective effects in the model	Gupta et al. (2023)
Citronellol	*In vivo* rat model by ROT	25 mg/kg/day, p.o., for 4 weeks, 30 min before ROT therapy	reducing oxidative stress, inflammation, and apoptosis. It also increased dopaminergic markers and influenced autophagy	Jayaraj et al. (2022)
Boswellia serrata gum extract	*In vivo* rat model by ROT	In Phase I: Boswellia extract was given at the dosage of 500 mg/kg/day, p.o., for 2 weeks	activating AMPK, protecting dopaminergic neurons, and reducing *a*-synuclein. It enhances neuroprotective molecules, lowers p-mTOR, and improves motor performance with increased dopamine levels	Shadfar et al. (2022)
		In Phase II: ROT was given at the dosage of 4 mg/kg/day, i.p., preceded by a treatment with Boswellia extract		
Kurarinone	*In vivo* mouse model by MPTP	5, 10, and 20 mg/kg/day, i.g., for 19 ays	Protecting dopaminergic neurons, reducing neuroinflammation, and acting as a potential soluble epoxide hydrolase inhibitor	Sun et al. (2022)
Urolithin A	*In vivo* mouse model by MPTP	20 mg/kg/day, i.p., for 7 days prior to MPTP injection	Reduced dopaminergic neuron loss, improved motor activity, and decreased neuroinflammation. Additionally, urolithin A induced mitophagy, restored mitochondrial function, and reduced NLRP3 inflammasome activation	Qiu et al. (2022)
Asarone	*In vitro* study using BV-2 cells activated with LPS	50 μM of *a*-asarone for 24 h	lowering of TNF-α levels	Qin et al. (2017)
	*In vivo study* using transgenic Tg (Apo-E:eGFP) zebrafish infected with *E. coli*	0.3–100 μM of *a*-asarone for 2 h	Inhibition of elongated changes in the morphology of M1 microglia generated by LPS by reducing MCP-1 expression	Cai et al. (2016)
			Reduction in the proportion of M1 microglia and the number of microglial tips	
Galangin	*In vitro* study using BV-2 cells treated with LPS	10–50 μM for 1 h	Suppression of inflammatory responses of M1 microglia by activating the Nrf2/CREB signaling pathway. Inhibition of excessive inflammatory activation of microglia	Choi et al. (2017)
	*In vivo* LPS-induced PD rat model	25, 50, and 100 mg/kg/day, p.o., starting 3 days before LPS injection for 28 days	Suppression of microglial M1 activation leads to lowered expression of pro-inflammatory chemicals, such as JNK, Akt, and NFκB communication pathways	Chen et al. (2017)
Baicalein	*In vitro* study using LPS and IFN-γ-induced BV-2 and RAW 264.7 cells	1–25 μM of baicalein, 60 min before the addition of LPS/IFN-γ	Suppression of iNOS expression by blocking the MAPKs and NFκB signaling pathways	Chen et al. (2004)
	*In vivo* MPP + neurotoxin rat model	10 and 30 mg/kg/day for 7 days	Protecting injured DA neurons by reducing microglia-induced inflammation and inflammasome activity	Hung et al. (2016)
α-Mangostin	*In vivo* study using a co-culture system of rat mesencephalic neurons and primary microglia	1, 10, and 100 nM for 24 h	Shielding DA neurons from neurotoxicity and prevention of the breakdown of IκBα and the translocation of NFκB p65 into the nucleus of M1-activated microglia	Hu et al. (2016)
Icariin	*In vitro* study using LPS activated- primary microglia	5, 10, and 50 μM for 30 min	Suppresses JNK, p38 MAPK, and NFκB signaling pathways, inhibits ROS.	Zeng et al. (2010)
	*In vivo* mouse model of PD	10 and 20 mg/kg/day, i.g., for 7 and 14 consecutive days	Modifies M1 microglia activation, reduces neuronal cell death, and inhibits NFκB phosphorylation	Wang et al. (2018)
Tenuigenin	*In vitro*	1, 2, and 4 μM for 24 h	Inhibits microglial M1 activation by activating the Nrf2/HO-1 pathway	Wang et al. (2017)
	*In vivo* study using MPTP -induced PD in male C57BL/6 J mice	25 and 50 mg/kg/day for 10 days before MPTP injection	Increases dopamine levels, suppresses the NLRP3 inflammasome and M1-activated microglia. Enhances the Nrf2/HO-1 pathway	Fan et al. (2017)
Nobiletin	*In vitro* study using LPS-stimulated BV-2 microglia	1–50 μM for 26 h	Inhibits pro-inflammatory cytokines by blocking MAPK phosphorylation and NFκB translocation	Cui et al. (2010)
	*In vivo* rat model	20 mg/kg, i.p., for 7 days	Protects dopamine neurons, suppresses M1 microglia, and lowers inflammatory cytokine release	Jeong et al. (2015)
Taurine	*In vivo* study using maneb and paraquat-induced PD mouse model	150 mg/kg/day, i.p., for 6 weeks	• Relieves dopaminergic neurotoxicity and motor impairments. Inhibits microglial activation, reduces *a*-syn protein accumulation, and suppresses M1 polarization and pro-inflammatory protein expression	Che et al. (2018)
			• Blocks NOX2 stimulation and interferes with p47phox translocation and NFκB process in M1 microglia	
Plantamajoside	*In vivo* and *in vitro* study using LPS-induced PD in male C57BL/6 mice and BV-2 cells	25 and 50 mg/kg/day in mice	• Reduced SN damage and decreased microglial cell overactivation	Guo et al. (2023)
		10, 20, and 40 μM in BV-2 cells	• Suppresses the activation of the HDAC2/MAPK pathway	
			• Reduced microglia polarization by inhibiting HDAC2	
Swertiamarin	*In vitro* and *in vivo* study using LPS-induced C6 glial cells and ROT-induced PD in mice	100 mg/kg/day, i.p., for 47 days in mice	• Reduces *a*-syn accumulation. Decreases pro-inflammatory cytokines in activated glial cells	Sharma et al. (2023)
		10–100 μg/mL in C6 glial cells	• Inhibits glial cell activation and *a*-syn overexpression	
			• Improves motor impairment and protects dopaminergic neurons	

#### 3.2.2.1 *a*-Cyperone

α-Cyperone, a major active compound of *Cyperus rotundus* L., which is often known as purple nutsedge or nut grass, is a common and widespread weed in tropical, subtropical, and temperate climates (Azimi et al., 2016). This plant has received a lot of attention for its medicinal potential, thanks to centuries of traditional medicine (Seo et al., 2001). It has been praised for its anti-arthritic, antidiarrheal, and antiplatelet characteristics, as well as its potential to treat numerous CNS illnesses such as epilepsy, depression, and inflammatory conditions (Jebasingh et al., 2012; Azimi et al., 2016). In a conducted investigation, researchers discovered that *a*-Cyperone at a dose of 10 mg/kg per day significantly improved motor function impairment, protected dopaminergic neurons, and counteracted the decrease of dopamine and its metabolites in a rat model of PD caused by lipopolysaccharide (LPS). Furthermore, *a*-Cyperone substantially reduced microglia activation and the production of many neuroinflammatory factors, including IL-6, IL-1β, TNF-α, iNOS, ROS, and COX-2 (B. Huang et al., 2023). The protective effects of *a*-Cyperone on microglia were explained by its ability to suppress neuroinflammation and oxidative stress, which was accomplished by activating the Nrf2/HO-1 pathway while simultaneously blocking the NFκB signaling pathway, according to meticulous molecular mechanism studies (B. Huang et al., 2023; Li et al., 2021). Furthermore, *a*-Cyperone aided in the overexpression of antioxidant enzymes such as glutamate-cysteine ligase catalysis, glutamate-cysteine ligase modifier, and nicotinamide quinone oxidoreductase 1 in microglia, adding to its neuroprotective characteristics (B. Huang et al., 2023; Jayaram and Krishnamurthy, 2021).

##### 3.2.2.2 Myrcene

Myrcene, sometimes known as *ß*-myrcene, is an abundant monoterpene found in a variety of plant species, including hops and cannabis (Surendran et al., 2021). Notably, myrcene is widely used in the food and beverage industries as a flavor and aroma enhancer, and it is also used as a food additive throughout the production process (Tyler, 1996; Surendran et al., 2021). Myrcene at a dose of 50 mg/kg, provided 30 min before rotenone (ROT) injections, demonstrated neuroprotective benefits in a mouse model of Parkinson’s disease (PD). ROT exposure resulted in the death of dopaminergic neurons, a decrease in antioxidant defenses, a rise in lipid peroxidation, and the activation of microglia and astrocytes. Pro-inflammatory cytokine levels were also raised, and the autophagy lysosomal pathway was disrupted, both of which contributed to dopaminergic neurodegeneration. Myrcene therapy, on the other hand, significantly restored antioxidant defenses, reduced lipid peroxidation, and pro-inflammatory cytokines, and reduced microglial and astrocyte activation. It also increased mTOR phosphorylation, which helped to restore neuronal homeostasis and autophagy-lysosomal activity. Surprisingly, myrcene treatment reduced *a*-synuclein expression, resulting in dopaminergic neuron preservation and rescue (Azimullah et al., 2023).

##### 3.2.2.3 Daphne genkwa flower

In East Asia, the flower buds of *Daphne genkwa* have been used as a traditional herbal medicine. Flavonoids derived from *D. genkwa* flower extract (GFE) have previously been investigated for their anti-inflammatory and antioxidant properties (Guo et al., 2020). Daphne GFE has also been shown to have anti-inflammatory properties as well as the ability to slow disease development in a PD animal model (Jiang et al., 2014; Guo et al., 2020). Researchers used neuroinflammation *in vivo* animal models produced by LPS to investigate the impact of Daphne GFE. C57BL/6 J mice aged eight to 9 weeks were randomly assigned to one of three groups: vehicle, LPS, or LPS + GFE. GFE was given at three different dosages (50, 100, and 200 mg/kg) orally over 3 days. LPS (4 mg/kg) intraperitoneal injection induced neuroinflammation 1 hour after the initial oral therapy. GFE inhibited the production of inflammatory factors (NO, iNOS, and TNF-α) in microglial and primary glial cells, resulting in significant anti-inflammatory and neuroprotective effects. It also stimulated phagocytosis in microglia and boosted the expression of neuroprotective markers (Arg-1 and brain-derived neurotrophic factor mRNA) in primary glial cells, emphasizing its potential as a PD treatment (Gupta et al., 2023).

##### 3.2.2.4 Citronellol

Citronellol (CT) is a monoterpene alcohol found in the essential oils of various plants that are used in cooking and traditional medicine (Santos et al., 2019). ROT was given once daily at a dose of 2.5 mg/kg for 4 weeks in a PD rat model. Concurrently, CT was administered orally once daily for 4 weeks at a dose of 25 mg/kg, 30 min before ROT therapy. CT had neuroprotective properties, suppressing ROS and lipid peroxidation while boosting antioxidant enzymes and decreasing brain inflammation. It also inhibited microglia and astrocyte activation, resulting in lower COX-2 and iNOS-2 expression. Furthermore, CT preserved and increased the levels of tyrosine hydroxylase in the substantia nigra pars compacta and striatum. Furthermore, it reduced apoptosis by decreasing Bax and *a*-synuclein levels while raising Bcl-2 and mTOR (Jayaraj et al., 2022).

##### 3.2.2.5 Methanolic extract of Boswellia serrata gum

For centuries, the resin of Boswellia species, namely, *Boswellia serrata* (Salai/Salai guggul), has been employed in religious, cultural, and medical traditions (Salama et al., 2023). The oleo gum resin is extracted from the tree’s trunk and comprises resin, essential oils, and polysaccharides. It is derived from many states in India and has historically been used in folk medicine to treat chronic inflammatory illnesses (Siddiqui, 2011). The researchers examined the neuroprotective properties of Boswellia serrata gum extract in PD in previous research. The extract activated AMPK and downstream neuroprotection pathways. *In vivo*, it protected nigrostriatal dopaminergic neurons while decreasing *a*-synuclein accumulation (Ameen et al., 2017).

During Phase I, to investigate whether Boswellia extract increases neuroprotective molecules, oral Boswellia extract (500 mg/kg/day) was given for 2 weeks, and Western blotting was used to identify neuroprotective compounds. In Phase II, to explore the neuroprotective effects of Boswellia extract on ROT neurotoxicity, four groups were studied: one as a control, one receiving oral Boswellia extract group, one receiving ROT (4 mg/kg/day, i. p.), and one receiving both Boswellia extract and ROT. Boswellia enhanced AMPK phosphorylation, decreased p-mTOR and p-α-synuclein in the striatum, and raised the expression of Beclin1 and brain-derived neurotrophic factor. Boswellia reduced dopaminergic neuron loss, microglial activation, and *a*-synuclein accumulation in ROT-injected rats, increasing striatal dopamine levels and motor performance (Shadfar et al., 2022).

##### 3.2.2.6 Kurarinone

Kurarinone is a naturally occurring prenylated flavanone isolated from *Sophora flavescens* (Huang et al., 2020). Kurarinone demonstrated promising therapeutic results in a PD mice model. It alleviated behavioral impairments and rescued dopaminergic neurons from 1-methyl-4-phenyl-1,2,3,6-tetrahydropyridine (MPTP)-induced neurotoxicity at dosages of 5, 10, and 20 mg/kg. In the substantia nigra and striatum, kurarinone retained neurotransmitters and tyrosine hydroxylase-positive cells. It also lowered neuroinflammation by blocking microglial activation via the NFκB signaling pathway. Kurarinone has been found to target soluble epoxide hydrolase, which is associated with neuroinflammation in PD (Sun et al., 2022).

##### 3.2.2.7 Urolithin A

Urolithin A is synthesized by gut bacteria from ellagitannin-rich food (Singh et al., 2021). Urolithin A therapy showed neuroprotective effects in a mouse model of PD. In mice, Urolithin A effectively reduced dopaminergic neuron loss, corrected behavioral impairments, and reduced neuroinflammation caused by MPTP (Ren et al., 2018). Further research indicated that Urolithin A induced mitophagy in BV2 cells (a type of microglial cell derived from C57/BL6 murine) that were exposed to LPS, restored mitochondrial function, and decreased the pro-inflammatory response. Furthermore, urolithin A significantly reduced NLRP3 inflammasome activation. To create the MPTP mouse model, mice were given 15 mg/kg MPTP intraperitoneally four times a day within a 2-h interval. Mice were given 20 mg/kg urolithin A intraperitoneally for 7 days before MPTP injection for urolithin A therapy. The rotarod, pole, and suspension tests all revealed significant impairment in motor activity after MPTP treatment. However, Urolithin A treatment dramatically corrected these motor abnormalities, suggesting the potential therapeutic advantages of Urolithin A in reducing motor impairments associated with PD (Qiu et al., 2022).

##### 3.2.2.8 Asarone

Asarone represents a phenylpropanoid found in plants such as Acorus and Asarum. It comes in two isomeric forms, *a* and *ß*. Because of its capacity to pass the BBB, *a*-asarone has significant neuroprotective effects. A dosage of 50 μM of *a*-asarone dramatically lowered TNF-α levels in tests using BV-2 cells activated with LPS (Qin et al., 2017). Interestingly, a lower quantity of *a*-asarone (3 μM) did not affect the generation of pro-inflammatory cytokines caused by LPS. However, it inhibited the elongated changes in the morphology of M1 microglia generated by LPS by lowering monocyte chemotactic protein-1 (MCP-1) expression. An *in vivo* study using transgenic Tg (Apo-E:eGFP) zebrafish infected with *E. coli* found that at the same dosage, *a*-asarone reduced the proportion of M1 microglia and the number of microglial tips. These findings emphasize *a*-asarone’s neuroprotective potential, specifically its potential to modulate inflammation and morphological alterations in microglia (Cai et al., 2016).

##### 3.2.2.9 Galangin

Galangin, a natural flavonol, predominantly exists in the rhizome of the therapeutic plant *Alpinia officinarum* (Zingiberaceae). This molecule was widely investigated and has been shown to stimulate PPAR-γ, a receptor involved in cellular process regulation. Galangin suppressed inflammatory responses of M1 microglia in tests employing BV-2 cells treated with LPS. This was accomplished by activating the Nrf2/cAMP response element-binding protein (CREB) signaling pathway at doses ranging from 10 to 50 μM (Choi et al., 2017). Furthermore, galangin showed encouraging results in an *in vivo* experimental rat model mimicking PD by inhibiting excessive inflammatory activation of microglia. Galangin also dramatically suppressed microglial M1 activation produced by LPS, lowering pro-inflammatory chemical expression. These effects were linked to changes in the JNK, Akt, and NFκB communication pathways. These findings emphasize galangin’s possible therapeutic role in modulating microglial inflammation, implying its potential as a therapy method for neurodegenerative disorders such as PD (Chen et al., 2017).

##### 3.2.2.10 Baicalein

Baicalein, which is derived from the root of *Scutellaria baicalensis* (Labiatae), has the potential to be used as a treatment for inflammatory conditions. Baicalein significantly suppressed the expression of iNOS in experiments involving BV-2 cells and primary microglia activated with LPS and IFN-γ. The effect mentioned was obtained by blocking the MAPKs and NFκB signaling pathways (Chen et al., 2004). Furthermore, baicalein displayed neuroprotective properties in a PD *in vivo* experimental model using MPP + neurotoxin. Baicalein protected injured DA neurons by reducing the activation of microglia-induced inflammation by reducing inflammasome activity. These findings emphasize baicalein’s medicinal potential in reducing inflammatory responses and protecting against neurotoxicity (Hung et al., 2016).

##### 3.2.2.11 *a*-Mangostin

α-Mangostin is a plant polyphenol produced from various parts of the *Garcinia mangostana*, a plant that has a xanthone core structure. The results of a recent *in vivo* study showed that *a*-Mangostin demonstrated a potent neuroprotective quality dependence on concentration. *a*-Mangostin at doses of 1, 10, and 100 nM effectively shielded DA neurons from neurotoxicity caused by microglial activation caused by *a*-syn in a co-culture system of rat mesencephalic neurons and primary microglia. The potential of *a*-Mangostin to prevent the breakdown of IκBα and the translocation of NFκB p65 into the nucleus of M1-activated microglia caused by *a*-syn was linked to its neuroprotective action. These data imply that *a*-Mangostin has potential as a neuroprotective drug against *a*-syn-mediated microglial neurotoxicity (Hu et al., 2016).

##### 3.2.2.12 Icariin

Icariin is a flavanol glycoside that has been prenylated and produced from several Epimedium species. It is the 8-prenyl derivative of kaempferol 3,7-O-diglucoside. In previous *in vitro* investigations, different concentrations of icariin (5, 10, and 50 μM) showed neuroprotective properties against the neurotoxicity induced by primary microglia activated with LPS. Additional research into the underlying mechanisms showed that icariin, in a manner dependent on the dosage used, suppressed the JNK, p38 MAPK, and NFκB signaling pathways by inhibiting the production of ROS and pro-inflammatory cytokines, including IL-6, TNF-α, and IL-1β in M1-activated microglia (Zeng et al., 2010). The neuroprotective characteristics of icariin were further investigated in a PD *in vivo* mouse model. Icarin, at doses of 0.01 and 0.1 μM, has been shown to modify M1 microglia activation produced by LPS/6-OHDA. This was accomplished by inhibiting NFκB p65 phosphorylation, which results in an indirect reduction in neuronal cell death (apoptosis) in primary rat midbrain neuroglia co-cultures (Wang et al., 2018).

##### 3.2.2.13 Tenuigenin

Tenuigenin is a bioactive terpenoid found naturally in the roots of *Polygala tenuifolia* (Polygalaceae). It has neuroprotective benefits such as antioxidant, anti-aging, and anti-inflammatory capabilities. An *in vitro* study using different doses of tenuigenin (1, 2, and 4 μM) revealed a dose-dependent decrease in LPS-induced microglial M1 activation. The activation of the Nrf2/HO-1 pathway was directly linked to this neuroprotective significance (Wang et al., 2017). Furthermore, by blocking DA breakdown, a higher dosage of tenuigenin (50 mg/kg) increased DA levels in mice’s striatum. Tenuigenin dramatically decreased DA neurodegeneration by suppressing the NLRP3 inflammasome and lowering the stimulation of M1-activated microglia. With its capacity to minimize microglial activation, inhibit the NLRP3 inflammasome, and enhance Nrf2/HO-1 pathway activation, tenuigenin has the potential to be a promising neuroprotective agent (Fan et al., 2017).

##### 3.2.2.14 Nobiletin

Nobiletin is a flavonoid compound in Tangerine peel (Citri reticulatae pericarpium). Numerous studies have demonstrated its antioxidant, anti-atherogenic, and anti-inflammatory qualities. Nobiletin administration at various doses (range from 1 to 50 μM) significantly suppressed the generation of pro-inflammatory cytokines, including IL-1β and TNF-α in a dose-dependent manner in a previous *in vitro* study using LPS-stimulated BV-2 microglia. According to detailed analysis, the underlying mechanism includes suppressing of phosphorylation of MAPKs and the translocation of NFκB into the nucleus (Cui et al., 2010). One notable feature of nobiletin is that it can penetrate the BBB and concentrate more in the brain than in peripheral organs. This distinct characteristic enables nobiletin to alleviate neuroinflammation and mitigate neuronal damage caused by M1-activated microglia (Singh et al., 2011). Furthermore, in a PD *in vivo* animal model, in which rats were injected with MPP+ in the middle forebrain bundle, using nobiletin (20 mg/kg, i. p., for 7 days) gave significant protection to DA neurons located in the SNpc. Nobiletin has also been shown to decrease M1 microglia activation, lowering inflammatory cytokines release (Jeong et al., 2015).

##### 3.2.2.15 Taurine

Taurine, a non-protein amino acid containing sulfur, is found in bile and several mammalian organs. The neuroprotective effect of taurine was explored in previous *in vivo* research employing a maneb and paraquat-induced model of PD. The researchers discovered that mice who received maneb and paraquat suffered from severe dopaminergic neurotoxicity and motor impairments, which were considerably relieved by taurine administration. Notably, taurine reduced the accumulation of *a*-syn, a characteristic protein linked with PD, in maneb and paraquat-intoxicated rats (Che et al., 2018). Additional studies into the mechanisms behind taurine’s neuroprotective properties found that Taurine can inhibit microglial activation caused by maneb and paraquat. Interestingly, reducing microglia activation abolished taurine’s neurological protective effects, demonstrating the importance of microglial activation in taurine-mediated neural protection. Taurine was also observed to decrease the M1 polarization of microglia and lower the expression levels of pro-inflammatory proteins induced by maneb and paraquat (Wang et al., 2021). Moreover, taurine inhibited the stimulation of NOX2, a critical enzyme implicated in oxidative stress. This was accomplished by interfering with the translocation of the cytosolic component p47phox and the NFκB process. NOX2 activation and NFκB signaling are essential in beginning and maintaining the inflammatory response of M1 microglia (Che et al., 2018).

##### 3.2.2.16 Plantamajoside

Plantamajoside, a natural product that comes from plantain seeds, has a wide range of biological activities, including anticancer, anti-inflammatory, and antioxidative features (Ravn et al., 2015). In an earlier *in vivo* research study, researchers used male C57BL/6 mice to induce a PD animal model by injecting LPS into the substantia nigra (SN) located in the midbrain region on the right side. The researchers discovered that Plantamajoside substantially alleviated the behavioral impairment caused by LPS in Parkinson’s disease rats. In addition, Plantamajoside has been shown to reduce SN damage produced by LPS and to decrease microglial cell over-activation in PD rats. Further investigation revealed that Plantamajoside exerted its effects in both PD mice and BV-2 cells by suppressing the activation of the histone deacetylase-2 (HDAC2)/MAPK pathway. Notably, Plantamajoside demonstrated its ability to reduce microglia polarization by inhibiting HDAC2 (Guo et al., 2023).

##### 3.2.2.17 Swertiamarin

Swertiamarin, a widely researched natural compound, has anti-inflammatory effects. It is obtained from Enicostemma littorale Blume and is classified as a secoiridoid glycoside (Fadzil et al., 2021). Swertiamarin has been demonstrated in the setting of PD to inhibit the accumulation of *a*-syn in a *Caenorhabditis elegans* model produced by 6-OHDA. A prior *in vivo* study aimed to assess swertiamarin’s anti-inflammatory action against LPS-induced activation of C6 glial cells and its neuroprotective benefits in a rat model of PD caused by intra-striatal ROT. Surprisingly, swertiamarin treatment reduced the levels of IL-1β, TNF-α, and IL-6 in LPS-induced C6 glial cell activation. In a mouse model of ROT-induced PD, swertiamarin (at a dosage of 100 mg/kg, i. p.) substantially inhibited the activation of microglial and astroglial cells in the SN. Furthermore, swertiamarin reduced ROT-induced *a*-syn overexpression in the striatum and the SN. Notably, swertiamarin improved motor impairment caused by ROT-induced neurotoxicity and reduced the loss of dopaminergic neurons in the nigrostriatal pathway. These data suggest that swertiamarin has tremendous promise as an additional treatment for PD (Sharma et al., 2023).

### 3.3 Huntington’s disease

#### 3.3.1 Role of microglia M1/M2 in Huntington’s disease

Huntington’s disease is a neurodegenerative condition with hereditary autosomal dominant traits. The main cause is a gene identified as huntingtin, which is found on the short arm p) of chromosome 4 and mutated to cause the disease (Gómez-Jaramillo et al., 2022). The CAG trinucleotide repeat expansion on chromosome 4 is the root cause of HD. Atypical involuntary motions, cognitive deterioration, and behavioral alterations are features of HD, in which the most apparent symptom is chorea (Gibson and Claassen, 2021). In contrast to normal control brains, considerable astrogliosis and microgliosis were found in the post-mortem brains of HD patients. According to a previous study, the density of microglia in the brains of HD patients fluctuated relative to the severity of neuronal loss (H. M. Yang et al., 2017). Furthermore, the extensive detection of M1 microglia significant biomarkers in HD brain suggests that M1 microglia may be important in the pathophysiology of HD. An alternatively activated M2 phenotype, however, has the potential to be neuroprotective, which contributes to HD recovery (Ji et al., 2018). Consequently, Pena-Altamira et al. concentrated on nutritional methods through the consumption of food-bioactive substances like carotenoids, phytosterols, and other substances that may affect microglial polarization, aid in neuron survival, and consequently lessen cognitive impairment associated with aging (Tang, 2018).

#### 3.3.2 Nutraceuticals that influence microglial activation in Huntington’s disease

**FIGURE 5:**
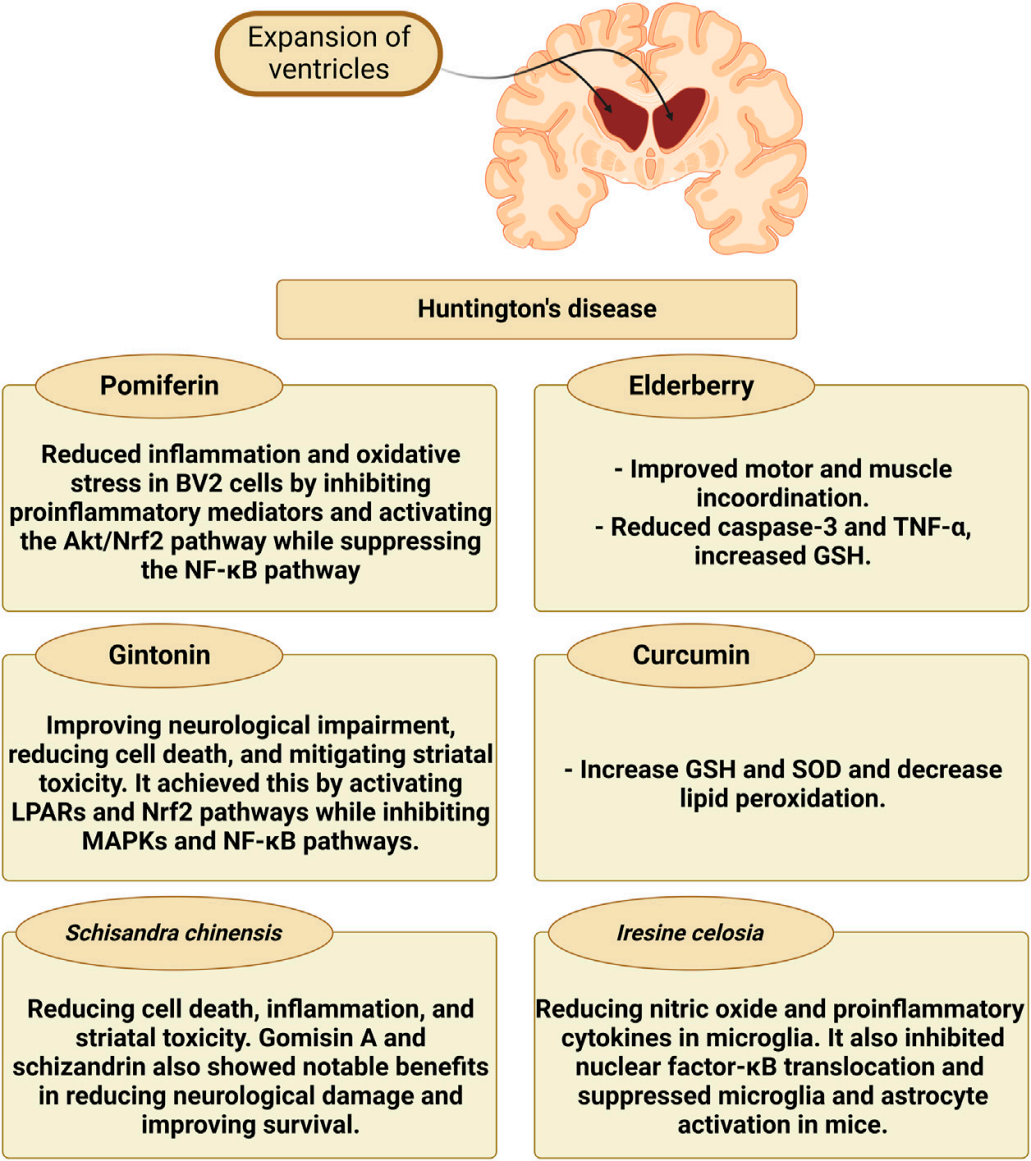
The effect of different nutraceuticals on microglial activation in Huntington’s disease.

The impact of different natural products and nutraceuticals on HD through regulation of M1/M2 microglia polarization is presented in Table 4 and Figure 5.

**TABLE 4:** The effects of different nutraceuticals on microglia in Huntington’s disease and Multiple sclerosis related models.

Compound	Model	Concentration/dose	Biological effects	References
Pomiferin	*In vitro* (BV2 cells)	0.25 μM, 0.5 μM, and 1 μM	Reduced inflammation and oxidative stress by inhibiting pro-inflammatory mediators and activating the Akt/Nrf2 pathway while suppressing the NFκB pathway	Zhao et al. (2022)
Gintonin	*In vivo* study using male adult C57BL/6NTac mice	25, 50, and100 mg/kg/day, p.o., for 5 days	Improving neurological impairment, reducing cell death, and mitigating striatal toxicity via activating lysophosphatidic acid receptors and Nrf2 pathways while inhibiting MAPKs and NFκB pathways	Jang et al. (2019)
*Schisandra chinensis*	*In vivo* mouse model by 3-NPA	75, 150, and 300 mg/kg/day, p.o. 30 min before 3-NPA therapy	• Reducing cell death, inflammation, and striatal toxicity	Kim et al. (2017)
			• Gomisin A and schizandrin reduced neurological damage and improved survival	
Iresine celosia	*In vitro* study using LPS-stimulated BV-2 cells	1, 10, and 100 μg/mL for 24 h in BV-2 cells	• Reducing NO and pro-inflammatory cytokines in microglia	Kim et al. (2019)
	*In vivo* study using LPS-injected mice	30 and 100 mg/kg/day, p.o., for 2 days before LPS treatment	• Inhibition of NFκB nuclear translocation and suppressing microglia and astrocyte activation in mice	
Elderberry	*In vivo* study using 3-NP-induced HD in rats	Oral diet containing 2% Elderberry for 8 weeks	• Improved motor and muscle coordination	Moghaddam et al. (2021)
			• Reduced caspase-3 and TNF-α, and increased GSH	
Curcumin	YAC128 HD mice	100 mg/kg/day for 8 weeks	Increase of the GSH and SOD, and decrease of lipid peroxidation	Sandhir et al. (2014), Gharaibeh et al. (2020)
	3-NPA-induced HD in rats	40 mg/kg/day, p.o., 1 hr. after 3-NPA, for 7 days		
Emodin	Female Sprague-Dawley rats and Guinea pigs	20 mg/kg/day, i.p., for 14 days	Reduction of inflammation and demyelination by increasing SIRT1 and PPAR-γ coactivator levels, and inhibiting NLRP3 inflammasome and microglia activation	Cui et al. (2023)
phenethyl ester of gallic acid (PEGA)	Dark Agouti (DA) rats	20 mg/kg/day, s.c., for 9 days	PEGA lowers the inflammatory potency of T cells and macrophages/microglia by inhibiting their ability to produce/release IL-17 and IFN-γ, as well as IL-6 and NO.	Stegnjai et al. (2022b)
Agathisflavone (FAB)	*Ex vivo* study in organotypic cerebellar slice model of myelination using P10-12 mice	5–50 Μm/day for 7 days *in vitro*	FAB affected microglial morphology, reduced soma size and ramification while enhanced the expression of the calcium binding protein Iba-1	Almeida et al. (2022)
Sinomenine	C57BL/6 mice	25 and 100 mg/kg/day, p.o., for 20 days	• Reduction in the levels of pro-inflammatory IL-1, IL-6, IL-18, TNF, and IL-17A alongside an increase in the levels of anti-inflammatory IL-10	Kiasalari et al. (2021)
			• Reduction in the levels of the inflammasome NLRP3, ASC, and caspase 1	
phenethyl ester of rosmarinic acid (PERA)	Dark Agouti (DA) rats	30 mg/kg/day, for 7 days	• Inhibition of the production of IFN-γ and IL-17, in immune cells derived from the CNS or lymph nodes	Stegnjai et al. (2022a)
			• Reduction of NO development in the CNS, lymph nodes, macrophages, and microglial cells	
α-Linolenic Acid–Valproic Acid Conjugate	N9 microglial cells	0.5 μM single dose	Reducing microglial production of the M1 pro-inflammatory marker iNOS	Rossi et al. (2020)
Resveratrol	*In vivo* female C57BL/6 J mouse model	3 mg/kg/day, intranasal	Reduction of TGF-β, IFN-γ, IL-1β, IL-6, and IL-17 in the brain/spinal cord	Zheng et al. (2023)
Huperzia serrata	Cuprizone-induced MS in Mice	0.2 mg/kg/day for 2 weeks	Upregulation of the mRNA expression of anti-inflammatory microglia-associated genes (iNOS and CD16) with downregulation of the mRNA expression of genes related to pro-inflammatory microglia	Zhang et al. (2022)
Icariin	*In vivo* C57BL/6 J female mouse model	7.5 and 15 mg/kg/day, p.o., for 28 days	• Reducing pathology of the myelin sheath in the white matter as well as neurons and microglia in the grey matter	Gao et al. (2023b)
			• Remission of neuroinflammation through inhibiting the activation of NLRP3 inflammasome	
Baicalein	*In vivo* C57BL/6 mouse model	150 mg/kg/day, i.g., from day 3 of immunization until day 20	• Reduced expression of iNOS and Iba-1	Ma et al. (2022)
			• Reduced phosphorylation of STAT1 in the spinal cord of the EAE group	

##### 3.3.2.1 Pomiferin

The main components of *Maclura pomifera* fruits are prenylated isoflavones, osajin, and pomiferin (Son et al., 2007). In a recent study exploring neurodegenerative conditions such as HD, researchers used many methods such as cck-8, LDH, quantitative PCR, and ELISA to explore the effects of pomiferin on BV2 cell inflammation. The results showed that pomiferin, at different concentrations (0.25 μM, 0.5 μM, and 1 μM), successfully decreased the production of NO, ROS, and pro-inflammatory mediators such as IL-6, TNF-α, iNOS, and COX-2 in BV2 cells (Zhao et al., 2022). Further research into pomiferin’s mechanism of action revealed that it activated the Akt/Nrf2 pathway while suppressing the NFκB pathway. These data show that pomiferin may have therapeutic potential for reducing neuroinflammation in neurodegenerative conditions via microglial pathways (Kobayashi and Yamamoto, 2005; Nguyen et al., 2009; Zhao et al., 2022).

##### 3.3.2.2 Gintonin

Gintonin is a glycolipid protein conjugated with lysophosphatidic acid that was originally extracted from Korean ginseng (Lim et al., 2022). Gintonin, administered orally at doses of 25, 50, or 100 mg/kg/day, showed promising results in reducing neurological impairment and striatal toxicity in an HD adult C57BL/6NTac adult mice model. After exposure to 3-nitropropionic acid (3-NPA), the striatum’s production of inflammatory mediators, microglial activation, and mitochondrial dysfunction was all decreased by pre-treatment with gintonin. Furthermore, gintonin suppressed MAPKs and NFκB signaling while activating lysophosphatidic acid receptors and Nrf2 signaling pathways (Jang et al., 2019). In mice with N171-82Q-mutant HTT overexpression, gintonin had neuroprotective effects by lowering cell death and mutant huntingtin protein aggregates in STHdh cells and improving neurological impairment. The beneficial effects of gintonin were lessened by pre-inhibiting lysophosphatidic acid receptors with Ki16425 (Wild and Tabrizi, 2014; Jang et al., 2019).

##### 3.3.2.3 Schisandra chinensis

A versatile traditional Chinese medicine; schisandra (*Schisandra chinensis*) is used for a variety of health problems, including respiratory, hepatic, gastrointestinal, and stress-related diseases. Additionally, it may help with menopausal symptoms, diabetes, polycystic ovarian syndrome, obesity, hepatitis, cognitive function, physical stamina, and as a preventative step for estrogen-dependent malignancies (Bokelmann, 2022). A mouse model of HD produced by 3-NPA was used to investigate *Schisandra chinensis* extract. The mice were given *Schisandra chinensis* extract at various doses (75, 150, or 300 mg/kg/day, orally) before or after 3-NPA therapy, Neuroprotective effects were investigated both before and after therapy, with pre-treatment being more beneficial. *Schisandra chinensis* extract decreased inflammatory markers, lesion area, cell death, and microglial activation. It inhibited the MAPK and NFκB signaling pathways while promoting the Nrf-2 pathway. Schizandrin and gomisin A, two *Schisandra chinensis* extract ingredients, reduced 3-NPA-induced brain damage and mortality (Kim et al., 2017).

##### 3.3.2.4 Iresine celosia

Iresine celosia is a cytochrome-flavoprotein with potent antioxidant properties (Porru et al., 2017). Neurological diseases, such as HD, are influenced by aberrant inflammatory responses in the central nervous system (Nayak et al., 2011). A recent research investigated managing neuroinflammation using natural therapies like Iresine celosia to address this. The researchers looked at the effects of Iresine celosia extract on LPS-stimulated BV2 cells in mouse models. NO and pro-inflammatory cytokines in microglia cells were considerably reduced by Iresine celosia extract at various doses (1–100 μg/mL) without causing any harm. In mice with neuroinflammation, it also prevented NFκB translocation and improved behavioral impairments (Kim et al., 2019).

##### 3.3.2.5 Elderberry

Sambucus spp. elderberries are cultivated in Europe, Asia, North Africa, and North America. Recent research indicates that elderberry assists in reducing some viral infections’ symptoms (Zakay-Rones et al., 2007). It has been established that anthocyanins, such as cyanidin-3-O-sambubioside and cyanidin-3-O-glucoside, and flavonoids, such as quercetin and rutin, are the antioxidative and anti-inflammatory substances present in the highest concentration in berries and flowers (Mikulic-Petkovsek et al., 2012). According to several additional studies, elderberry possesses various neuroprotective qualities (by reducing microglial activation), which can lower the loss of neuronal cells (Jiang et al., 2015; Zielińska-Wasielica et al., 2019).

To demonstrate the extreme nature of neuro-inflammatory processes, immunohistochemistry staining for the microglia marker (Iba-1) was performed in recent investigations. The results showed that rats given elderberry had significantly decreased microgliosis. The study concluded that elderberry’s antioxidant action increased GSH concentration and reduced ROS production in the tissue that had been damaged. Additionally, by reducing microglia’s production of TNF-α, this study sheds new illumination on using elderberry as neuroprotective medicine to treat HD by improving neuron survival due to microglial inactivation (Moghaddam et al., 2021).

##### 3.3.2.6 Curcumin

According to a previous *in vivo* study in YAC128 HD mice, solid lipid nanoparticles of curcumin reduced HD-like neurodegeneration. By boosting glutathione levels and reducing superoxide dismutase activity, solid lipid nanoparticles of curcumin greatly reduced protein carbonyl formation, lipid peroxidation, ROS levels, and mitochondrial swelling (Gharaibeh et al., 2020). Additionally, in a 3-NPA-induced HD model in rats, curcumin therapy is believed to improve cognitive and motor abilities, regain succinate dehydrogenase activity, and lessen oxidative stress (Sandhir et al., 2014). Curcumin can cross across the BBB because it is lipid soluble. It then blocks the activation of microglia by decreasing the expression of iNOS. Curcumin suppresses cytokines release and oxidative stress and lowers NO generation, and the related signaling pathways, which has an anti-inflammatory effect on microglia. In addition, curcumin inhibits apoptosis, PI3k/Akt and iNOS, lipoxygenase, and COX-2, and induces activation of HO-1, Nrf-2, and the antioxidant response element mechanism in neuronal cells as well as microglia (Ghasemi et al., 2019). Furthermore, curcumin also rescues downregulated molecular chaperones in HD, including Hsp40 and Hsp70, which have superior roles in the disease progression (Suzuki et al., 2023).

### 3.4 Multiple sclerosis

#### 3.4.1 Role of M1/M2 in multiple sclerosis

The most prevalent autoimmune disease that results in non-traumatic neurological damage in young adults is MS. Inflammation with demyelination and astroglial growth (gliosis) and neurodegeneration are the two pathological hallmarks of MS (Hauser and Cree, 2020). In the early stages of MS, axonal damage is detected. Acute, inflammatory lesions consisted of more transected or swollen axons than chronic plaques. It has been hypothesized that inflammation plays an essential role in the ongoing neurodegeneration in MS because of the beneficial association between axonal inflammation and structural modifications (Correale, 2014). It has been observed that peripheral inflammatory cells infiltrate into the CNS tissues, presumably because of BBB breaking down in the sick condition. The majority of the peripheral inflammatory cells that are infiltrating are macrophages and microglia, followed by CD^4+^ T cells, whereas the CNS consists of just a few CD^8+^ T cells, B cells, and plasma cells (Chu et al., 2018). Microglial activation in early lesions with a proliferation of macrophages in seriously injured tissue has also been associated with active demyelination and neurodegeneration (Correale, 2014). Using a panel of markers that distinguish M1-or M2-type macrophages/microglia, it was discovered that M1-type differentiated cells accumulated preferentially around the lesion edge, suggesting a vital purpose for these cells in the development of MS lesions (Ja et al., 2020).

The presence of myelin and axonal remains, the elevated levels of major histocompatibility complex-II and co-stimulatory molecules, and the release of several inflammatory and neurotoxic mediators through activated macrophages and microglia in MS and experimental autoimmune encephalomyelitis (EAE) lesions are all consistent findings. Additionally, studies using bone marrow chimeras demonstrated that the activation and proliferation of microglia preceded the beginning of EAE and that suppressing their activation prevents the growth and maintenance of inflammatory lesions in the CNS. It is important to observe that MS patients have active microglia clusters dispersed throughout their generally normal-looking white matter (Bogie et al., 2014).

#### 3.4.2 Nutraceuticals that influence microglial activation in multiple sclerosis

**FIGURE 6:**
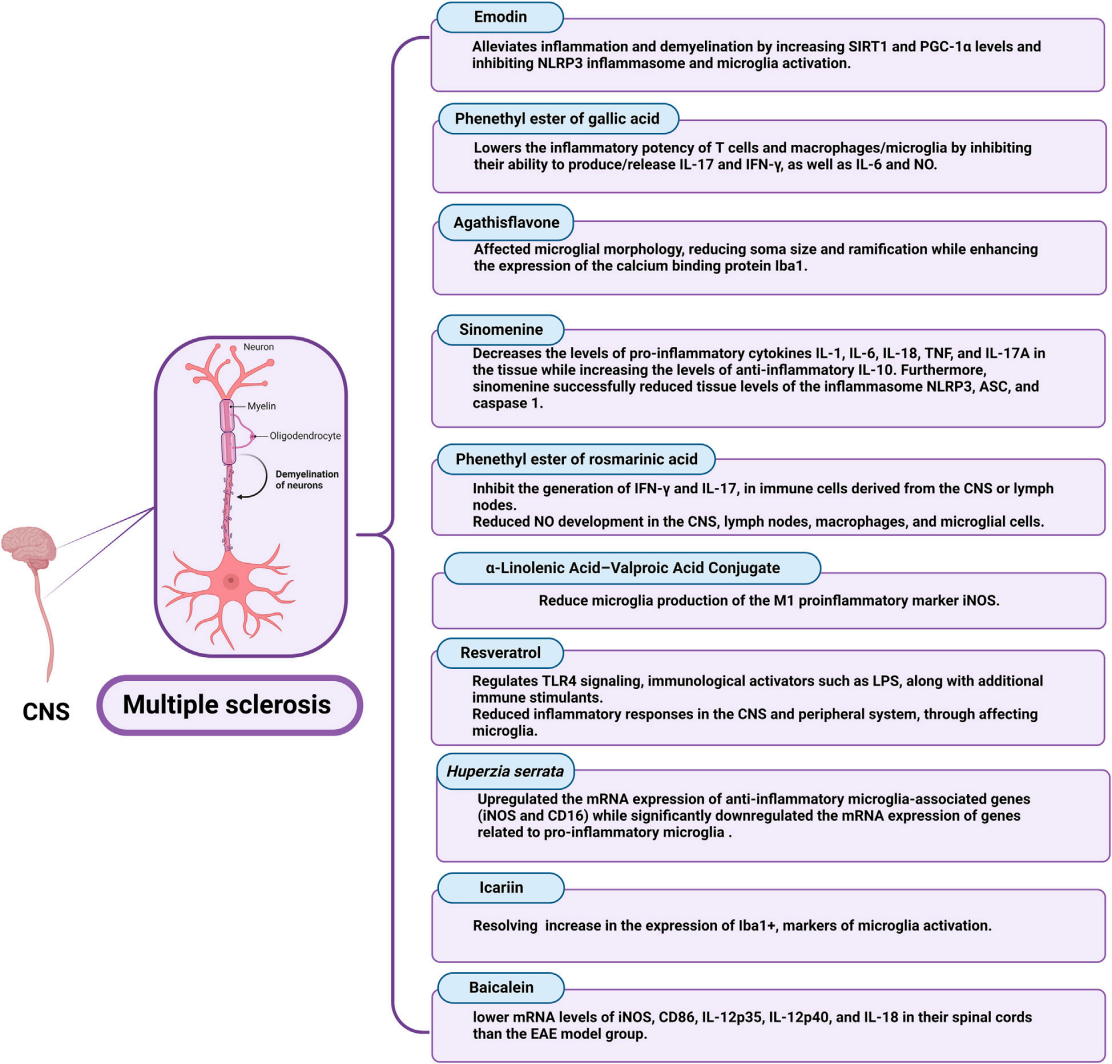
Nutraceuticals that influence microglial activation in Multiple Sclerosis.

The impact of different natural products and nutraceuticals on MS through regulation of M1/M2 microglia polarization is presented in Table 4 and Figure 6.

##### 3.4.2.1 Emodin

Emodin (1, 3, 8-trihydroxy-6-methylanthraquinone), a chemical derived from herbs such as rhubarb, has a neuroprotective effect (Zhu et al., 2019). Emodin has been shown in studies to have a variety of pharmacological actions, including antioxidant, immunomodulatory, and anticancer activities involving autophagy and apoptosis (Mitra et al., 2022). Emodin suppresses the action of the casein kinase 2 protein kinase. In EAE, which is an inflammatory, autoimmune demyelinating condition that affects rodents’ CNS and is clinically and pathologically similar to MS in humans, systemic casein kinase 2 inhibition or its reduction in CD4^+^ T cells has neuroprotective effects (Gibson and Benveniste, 2018). *In vitro* investigations with female Sprague-Dawley rats and guinea pigs indicated that emodin reduced the severity of EAE; also, the expression levels of NLRP3 signaling pathway components, COX-2, TNF-α, and IL-6 were lowered in the EAE group treated with emodin (Cui et al., 2023). COX-2 is secreted by immune cells, and its greater expression could indicate immune cell activity and increased inflammation (Nitric et al., 2005). Treatment with emodin restored iNOS and NO production in the research, which is consistent with the benefits of Emodin treatment found in other animal models of disease, suggesting the therapeutic effects of emodin in EAE rats and the probable involvement of the NLRP3 signaling system (Hu et al., 2021). Furthermore, produced inflammatory cytokines can encourage the aggregation and activation of resting microglia, which in response produce inflammatory cytokines, creating a vicious cycle (Wu et al., 2021). However, they discovered that emodin therapy decreased microglial aggregation and activation in EAE mice and lowered inflammatory levels in BV2 cells, resulting in neuroprotective advantages. Nonetheless, they discovered that EAE rats had lower levels of sirtuin 1 and Peroxisome proliferator-activated receptor-gamma coactivator expression and higher levels of NLRP3 inflammasome component expression than EAE + emodin rats. In EAE rats, emodin therapy resulted in elevated sirtuin 1 and Peroxisome proliferator-activated receptor-gamma coactivator levels as well as symptom relief (Cui et al., 2023).

##### 3.4.2.2 Gallic acid

Gallic acid (GA), a secondary metabolite abundant in many plants, nuts, and fruits, has been associated with anti-inflammatory activities. GA’s anti-inflammatory activities have been attributed to its capacity to interfere with MAPK and NFκB signaling, restricting immune cell activation and effector qualities (Bai et al., 2021). The phenethyl ester of gallic acid (PEGA) was developed to enhance gallic acid bioavailability and consequently its therapeutic potential. PEGA has been demonstrated to affect the inflammatory activities of T-cells and macrophages/microglia in an *in vivo* study of its effects on encephalitogenic cells (Stegnjai et al., 2022b) PEGA lowered the inflammatory potency of T-cells and microglia by inhibiting their ability to produce or release IL-17 and IFN-γ, as well as IL-6 and NO. Additionally, it significantly limits ongoing inflammatory responses against the CNS while also alleviating EAE. The reported effect of PEGA on T cells in the lymph nodes and the spinal cord’s ability to produce IFN-γ and IL-17 is particularly relevant for its advantageous effect on EAE.

Furthermore, limiting NO production by immune cells in the CNS is critical for PEGA’s beneficial impact on EAE. Much of the CNS tissue death during neuroinflammation is caused by the harmful effects of NO and its metabolite peroxy-nitrite (Spasojevic and Miljkovic, 2013). Furthermore, macrophage/microglia production of inflammatory cytokines TNF-α and IL-6 have been related to the etiology of CNS autoimmunity (Shemer and Jung, 2015; O’Loughlin et al., 2018). As a result, the suppressive impact of PEGA on the release of IL-6 in macrophages/microglia and TNF-α in microglia may contribute to the agent’s amelioration of EAE. PEGA’s inhibitory effects on NO, TNF-α, and IL-6 are consistent with the previously reported effects of GA and GA-like substances on immune cells in both *in vivo* and *in vitro* studies (Stegnjai et al., 2022b).

##### 3.4.2.3 Flavonoids

Flavonoids are plant-derived natural chemicals that have powerful anti-oxidant, anti-inflammation, and anti-neurodegeneration properties (Lima et al., 2016). Agathisflavone (FAB) is a bioflavonoid isolated from Poincianella pyramidalis that has minimal toxicity and a variety of biological activities, including anti-inflammatory, neuroprotective, and neurogenic properties, as well as the ability to reduce astrogliosis and microgliosis after lysolecithin-induced demyelination (Almeida et al., 2020; dos Santos Souza et al., 2018). Proteolipid protein, a cholesterol-associated protein with important roles in myelin membrane intracellular trafficking; myelin basic protein, is a key protein involved in myelin compaction. Meanwhile, cyclic nucleotide phosphodiesterase is an important protein involved in the maintenance of a normal inner tongue during the myelination of small-diameter axons. The absence of any of these proteins results in significant myelin sheath alterations and/or demyelination (Snaidero et al., 2017). In the previous investigations at doses above 5 μg, FAB enhances the amount of myelin basic protein immunolabelling of NF^+^ axons in postnatal cerebellar slice cultures. At these doses, however, the numbers of oligodendrocyte progenitor cells and oligodendrocytes were unaltered, showing that FAB enhanced the degree of myelination per oligodendrocyte rather than increasing their overall number (Almeida et al., 2022). Although, they demonstrate how FAB affected microglial morphology, reducing soma size and ramification while enhancing the expression of the calcium-binding protein Iba1. On the other hand, FAB did not influence microglial connections with oligodendrocytes, which may be essential to the release of trophic substances onto oligodendrocytes (Ioannidou et al., 2014; Domingues et al., 2016). FAB modulates microglia, which is consistent with *in vitro* data that FAB is anti-inflammatory and may shift microglia to a less active state (Almeida et al., 2022).

##### 3.4.2.4 Sinomenine

An alkaloid called Sinomenine exists in the roots of *Sinomenium Acutum* and has previously been used to treat arthritis. Sinomenine also has anti-inflammatory and immunosuppressive properties, as well as potential benefits for treating neurological diseases (Hong et al., 2022). Recently, the natural alkaloid sinomenine can significantly reduce neuroinflammation and demyelination in the spinal cord of a myelin oligodendrocyte glycoprotein 35–55 immune-mediated EAE model of MS. This was shown by partial suppression of microglial and astrocytic activation and pro-inflammatory cytokines, as well as by restoration of anti-inflammatory cytokines and improvement of motor functions. Inflammasome NLRP3 and the associated caspase-1 pyroptotic pathway were additionally suppressed by sinomenine (Kiasalari et al., 2021). Furthermore, abnormal alterations in pro-inflammatory and anti-inflammatory cytokines play critical roles in the pathophysiology of autoimmune illnesses such as MS and EAE (Fontes et al., 2017; Ma et al., 2017). According to the findings, sinomenine can reduce the tissue levels of TNF-α, IL-6, IL-18, IL-1, and IL-17, while suitably elevating anti-inflammatory IL-10 in the myelin oligodendrocyte glycoprotein-immunized EAE group. Consistent with these findings, sinomenine has been shown to reduce pro-inflammatory mediators and downregulate oxidative stress in a mouse model of ankylosing spondylitis (Dong et al., 2019), and sinomenine has been shown to protect against inflammation-related pain by reducing inflammatory mediators via p38-MAPK/NF-B signaling (Yuan et al., 2018).

##### 3.4.2.5 Rosmarinic acid

Rosmarinic acid is a polyphenolic chemical found in abundance in Lamiaceae herbs. It has been shown to have powerful anti-inflammatory effects both in *vitro* and *in vivo* studies (Luo et al., 2020). Rosmarinic acid has been demonstrated to block the T helper-17 axis in psoriasis-like illness in mice, as well as dendritic cell antigen-presenting potency *in vitro* (Zhang et al., 2021). These rosmarinic acid actions are crucial for MS therapy because T-helper-17 cells are among the most significant pathogenic cells in this disease (Murúa et al., 2022). Rosmarinic acid’s *in vivo* effects are limited because its polar carboxylic acid group inhibits the compound’s ability to permeate cellular membranes. To increase its bioavailability, lipophilic ester- and amide-derivatives of rosmarinic acid (Gerogianni et al., 2018), such as phenethyl ester of rosmarinic acid (PERA), were synthesized (Stegnjai et al., 2022a). A recent study showed that PERA significantly lowers the clinical course of EAE. This effect corresponds to a decrease in NO in both the CNS and the peripheral immune compartment. In addition, PERA inhibits IFN-γ and IL-17 production by popliteal lymph node cells and spinal cord immune cells in an *in vitro* study (Stegnjai et al., 2022a). NO, and its product peroxynitrite is a significant participant in the destruction of CNS tissue during neuroinflammation (Cross et al., 1997). Indeed, significant amounts of NO released locally within the CNS were found to be harmful in DA rats with EAE (Miljković et al., 2011; Petković et al., 2013). Thus, the reported NO-inhibiting actions of PERA on macrophages and microglia are extremely relevant to the PERA model’s positive benefits. Furthermore, PERA’s inhibitory effect on microglia release of the inflammatory cytokines; TNF-α and IL-6, is likely to contribute to the compound’s demonstrated positive effects in EAE, as microglia production of these cytokines has been linked to the etiology of CNS autoimmunity (Shemer and Jung, 2015; O’Loughlin et al., 2018). These PERA effects are consistent with the previously documented effects of rosmarinic acid and its related compounds on NO, TNF-α, and IL-6 *in vivo* and *in vitro* (Stegnjai et al., 2022a).

##### 3.4.2.6 *a*-Linolenic acid–valproic acid conjugate

Rossi et al. developed medications that incorporated valproic acid and *a*-linolenic acid, either linked or conjugated. By using N9 microglial cells that have been treated with 100 ng/mL LPS, they found that diamide conjugate and ethanolamide conjugate significantly reduced microglia production of the M1 pro-inflammatory marker iNOS at low concentrations (0.5 μM) (Rossi et al., 2020).

##### 3.4.2.7 Resveratrol

Resveratrol regulates TLR4 signaling, and immunological activators such as LPS, along with additional immune stimulants (Malaguarnera, 2019). The NF-kB pathway, COX-2 pathway, TLR expression, and activation of the NLRP3-inflammasome are all inhibited by resveratrol, which mediates these effects and promotes the production of anti-inflammatory M2 macrophages (Moudgil and Venkatesha, 2023). As a result, an approach being developed to treat MS involves intranasal delivery of RAW-Exo formulation with added resveratrol had led to improving the clinical progression of MS in an *in vivo* study, and greatly reduced inflammatory responses in the CNS and peripheral system, by affecting microglia (Zheng et al., 2023).

##### 3.4.2.8 Huperzia serrata

*Huperzia serrata* has been demonstrated to play a role in several animal models of neurological illnesses, as well as to alleviate cognitive impairment, diminish neuroinflammation, improve neuroprotection by boosting cortical inhibition, and enhance neuroprotection at low dosages. Multiple *in vivo* and *in vitro* research investigations have demonstrated that *H. serrata* may directly influence microglia to decrease CNS inflammation. It was discovered that the hippocampus and corpus callosum both had considerably fewer microglia following *H. serrata* therapy. In the *H. serrata*-treated mice, there were significantly fewer microglia branch endings and shorter overall branch lengths, indicating that *H. serrata* could effectively control microglia activation (H. Zhang et al., 2022). It has been proven that the pathophysiology of MS corresponds to the microglia-associated mRNA of pro-inflammatory and anti-inflammatory genes. Quantitative RT-PCR was applied to assess the mRNA expression of several pro-inflammatory and anti-inflammatory genes in the microglia to examine the potential role of *H. serrata* in cuprizone-induced inflammation. According to the study’s results, *H. serrata* significantly upregulated the mRNA expression of anti-inflammatory microglia-associated genes (iNOS and CD16) while significantly downregulating the mRNA expression of genes related to pro-inflammatory microglia (Zhang et al., 2022).

##### 3.4.2.9 Icariin

Icariin is a naturally present flavonoid glucoside that has been identified in the family of Epimedium that is frequently utilized in traditional Chinese medicine (Gao et al., 2023b). Degeneration of the white matter is commonly associated with MS. Comprehensive examination of the neuropathology also indicates severe pathology in the cortical and deep grey matter. Examination for NeuN expression in, the neuronal marker protein that is used to estimate neuronal density in the brain, determined the effect of Icariin on the grey matter. In the hippocampus and cortex of EAE mice modeled *in vitro*, there were considerably fewer NeuN + neurons than in the control group (Gao et al., 2023b). Glial cell activation is a hallmark of neuroinflammation. In the hippocampus and cortex of EAE animals, there was an apparent increase in the expression of Iba1+, markers of microglia activation, and the microglia’s structure was investigated to be longitudinally branching. Importantly, these defects almost completely resolved in mice administered high doses of Icariin (Gao et al., 2023b).

##### 3.4.2.10 Baicalein

The primary flavonoid found in *S. baicalensis* Georgi, a traditional Chinese medicine, is called baicalein. It exhibits antioxidant, anti-inflammatory, anticancer, antiviral, and neuroprotective pharmacological activities. (Ma et al., 2022). Iba-1 is an indication of activated macrophages and microglia. Arg1 is a polarized marker for M2 microglia/macrophages, while iNOS is a marker for M1 microglia/macrophages. In recent *in vivo* and *in vitro* studies, the number of microglia and macrophages that were both iNOS- and Iba-1-positive decreased considerably after baicalein therapy. In addition, resulting from baicalein treatment, Iba-1, and iNOS expression significantly lowered in comparison to the EAE model group. Nonetheless, qRT-PCR was applied to identify the expression of M1 inflammatory markers in each group’s mouse spinal cord. The outcomes demonstrated that animals receiving baicalein or dexamethasone had significantly lower mRNA levels of iNOS, CD86, IL-12p35, IL-12p40, and IL-18 in their spinal cords than the EAE model group (Ma et al., 2022).

## 4 Clinical trials on nutraceuticals in neurodegenerative diseases

Concerning human data, a few randomized clinical trials have been conducted for neurodegenerative disorders after natural product administration. Only a small number of clinical research has looked at the impact of curcumin on human cognitive performance in AD, compared with animal studies. The findings of these investigations are contradictory. Some studies show no cognitive enhancing benefits of curcumin (Baum et al., 2008; Ringman et al., 2012), whilst other studies suggested a positive effect of curcumin on cognition (Cox et al., 2015; Rainey-Smith et al., 2016; Small et al., 2018). Several investigations reveal protective mechanisms of curcumin against cognitive impairment, similar to animal research (Baum et al., 2008; Small et al., 2018). However, neuroimaging suggests that curcumin reduces Aβ deposits in the brain (Small et al., 2018). Results regarding Aβ reduction are ambiguous because most peripheral measurements, such as plasma, serum, and CSF levels, have not detected significant changes in Aβ or tau levels between curcumin and placebo (Baum et al., 2008; Ringman et al., 2012). Unfortunately, only one study by Baum et al. published measures of oxidative stress biomarkers, while no other study reported measurements of inflammatory biomarkers, even though these are the principal targets of curcumin and have demonstrated significant improvement in animal research (Baum et al., 2008). Meanwhile, data from human studies of curcumin in Huntington’s disease is lacking, and clinical trials should be strongly promoted in this area.

Regarding resveratrol, a phase II clinical trial evidenced the safety and well-tolerated effects of 500 mg orally once daily resveratrol on mild to moderate AD patients for 52 weeks, with no observed effects on AD biomarkers (Turner et al., 2015). However, decreased MMP 9 in CSF, controlled neuro-inflammation, adaptive immunity generation, and attenuated cognitive decline were confirmed in further investigation (Moussa et al., 2017). Meanwhile, another randomized placebo-controlled study on a small number of AD patients that received resveratrol (5 mg/day), showed less cognitive deterioration. In contrast, a dose of 200 mg/day of RV for 26 weeks did not experience any appreciable improvements in verbal memory function in 60 elderly individuals (Huhn et al., 2018).

Furtherly, in patients with AD, *H. serrata* appears to positively impact the restoration of cognitive function, performing everyday tasks, and overall clinical assessment (Yang et al., 2013). In addition, according to a clinical investigation, taurine has a positive effect on PD patients’ motor symptoms, and its levels in their plasma were decreased (Zhang et al., 2016). A prospective cohort study showed that *a*-Linolenic acid is associated with improved magnetic resonance imaging lesions activity in 87 multiple sclerosis patients (Bjornevik et al., 2019).

## 5 Conclusion

In our review, we emphasized the role and polarization control of microglia in the pathophysiology of neurodegenerative disorders and briefly introduced the function and phenotype of microglia to explore prospective therapeutic approaches. Transcription factors, receptors, and cytokines are merely a few examples of the numerous categories that modulate microglia polarization from M1 to M2. TNF-α, ILs, CXCLs, ROS, and NO, which have pro-inflammatory and neurotoxic effects, are released when the M1 phenotype is activated by IFN- γ, LPS, and TLR pathways. Meanwhile, M2 microglia are activated by different types of ILs to release abrineurin and anti-inflammatory cytokines, such as TGF-β, which suppress inflammatory responses and have neuroprotective effects. NcRNAs, such as miRNA-155, miR-23b-3p, and circHIPK3, seem to represent key factors in shifting microglia from M1 to M2. Consequently, inhibiting M1 microglia and promoting M2 microglial activation is required for neurodegenerative cure. In AD, curcumin, aromatic-turmerone, caeminaxin A, myricetin, aurantiamide, 3,6′-disinapoylsucrose, and resveratrol restored the normal balance of M1/M2 markers in microglia, which was also achieved by plant extracts of *D. cochinchinensis* stemwood and *O. majorana* L. Microglia-mediated apoptosis was prohibited because of andrographolide, sulforaphane, triptolide, xanthoceraside, and piperlongumine inhibition of Aβ-induced microglial M1 activation. In PD, urolithin A, kurarinone, asarone, galangin, baicalein, and mangostin hindered ROS and pro-inflammatory cytokines in M1-activated microglia. Further, icariin and tenuigenin suppressed microglial neurotoxicity via inhibiting NLRP3 inflammasome. Likewise, citronellol, myrcene, *a*-cyperone, nobiletin, taurine, plantamajoside, and swertiamarin showed neuroprotective effects in Parkinson’s. Similarly, elderberry, curcumin, iresine celosia, *Schisandra chinensis*, gintonin, and pomiferin showed promising results in improved symptoms in HD patients. In MS, linolenic acid, resveratrol, *H. serrata*, icariin, and baicalein are potent inhibitors of microglial activation, in addition to emodin, PEGA, FAB, sinomenine, and PERA, which all showed improved disease outcomes. Therefore, additional studies are required to resolve the significant enigma surrounding microglia polarization. It is important to note that these studies in the particular context of neurodegenerative illnesses clearly demand additional research. Moreover, several regulators of the microglia phenotype, which connects cytokine and signal pathways, may be linked to one another. It is still unknown how other risk genes affect microglia polarization in neurodegenerative disorders. Finally, the available findings encourage incorporating the natural product in treatment guidelines for neurodegenerative diseases, but more proof from randomized controlled studies on their effectiveness is highly warranted.

## Figures and Tables

**Table:** 

**Aβ**	amyloid-beta
**AD**	Alzheimer’s disease
**Akt**	Protein kinase B (PKB)
**AMPK**	AMP-activated protein kinase
**APP**	amyloid precursor protein
**Arg-1**	arginase-1
**ASC**	apoptosis-associated speck-like protein containing a caspase recruitment domain
**α-syn**	alpha-synuclein
**BBB**	Blood-Brain Barrier
**Bcl-2**	stands for B-cell lymphoma 2
**cAMP**	cyclic adenosine monophosphate
**cGAS**	cyclic GMP-AMP synthase
**circRNA**	circular RNA
**CNS**	central nervous system
**COX-2**	cyclooxygenase-2
**CREB**	cyclic AMP response element-binding protein
**CSF**	cerebrospinal fluid
**CT**	Citronellol
**CXCL**	C-X-C motif chemokine ligand
**DA**	dopamine
**DAMP**	damage-associated molecular pattern
**EAE**	experimental autoimmune encephalomyelitis
**eRNA**	enhancer RNA
**FAB**	Agathisflavone
**GA**	Gallic acid
**GFE**	genkwa flower extract
**GM-CSF**	granulocyte macrophage-colony stimulating factor
**GSH**	reduced glutathione
**HD**	Huntington’s disease
**HDAC2**	Histone deacetylase 2
**HO-1**	heme oxygenase-1
**IFN-γ**	interferon-gamma
**IL**	interleukin
**iNOS**	inducible nitric oxide synthase
**IRF3**	interferon regulatory factor 3
**JAK**	Janus kinase
**JNK**	c-Jun N-terminal kinase.
**Iba-1**	ionized calcium-binding adapter molecule 1
**IκBα**	I kappa B alpha
**lncRNA**	long non-coding RNA
**LBs**	Lewy bodies
**LPS**	lipopolysaccharide
**MALAT1**	metastatic-associated lung adenocarcinoma transcript 1
**MAPK**	mitogen-activated protein kinase.
**MIP-1β**	macrophage inflammatory proteins-1 beta
**miRNA**	microRNA
**MPP+**	1-methyl-4-phenylpyridinium ion
**MPTP**	1-methyl-4-phenyl-1,2,3,6-tetrahydropyridine
**MS**	multiple sclerosis
**mTOR**	Mammalian Target of Rapamycin
**Myd88**	myeloid differentiation factor 88
**NFκB**	nuclear factor kappa B
**NLRP3**	nucleotide-binding domain, leucine-rich–containing family, pyrin domain–containing-3
**NO**	nitric oxide
**NOD**	nucleotide-binding oligomerization domain
**NOX2**	NADPH oxidase 2
**Nrf2**	nuclear factor erythroid 2-related factor 2
**3-NPA**	3-nitropropionic acid
**nt**	nucleotides
**6-OHDA**	6-hydroxydopamine PATs promoter-associated transcripts
**PD**	Parkinson’s disease
**PEGA**	phenethyl ester of gallic acid
**PERA**	phenethyl ester of rosmarinic acid
**piRNA**	PIWI-interacting RNA
**PPAR-γ**	proliferator-activated receptor gamma
**PS1**	presenilin-1
**ROS**	reactive oxygen species
**ROT**	rotenone
**siRNA**	small-interfering RNA
**sncRNA**	small non-coding RNA
**SN**	substantia nigra
**SNpc**	substantia nigra pars compacta
**SNPs**	single nucleotide polymorphisms
**STAT1**	signal transducer and activator of transcription 1
**STING**	stimulator of interferon genes
**SWE**	Swertiamarin
**Tg (Apo-E:eGFP)**	Transgenic Apolipoprotein E: enhanced Green Fluorescent Protein
**TGF-β**	transforming growth factor β
**TLR**	toll-like receptor
**TNF-α**	tumor necrosis factor-alpha
